# Compacting a synthetic yeast chromosome arm

**DOI:** 10.1186/s13059-020-02232-8

**Published:** 2021-01-04

**Authors:** Zhouqing Luo, Kang Yu, Shangqian Xie, Marco Monti, Daniel Schindler, Yuan Fang, Shijun Zhao, Zhenzhen Liang, Shuangying Jiang, Meiwei Luan, Chuanle Xiao, Yizhi Cai, Junbiao Dai

**Affiliations:** 1 CAS Key Laboratory of Quantitative Engineering Biology, Guangdong Provincial Key Laboratory of Synthetic Genomics and Shenzhen Key Laboratory of Synthetic Genomics, Shenzhen Institute of Synthetic Biology, Shenzhen Institutes of Advanced Technology, Chinese Academy of Sciences, Shenzhen, 518055 China; 2 Key Laboratory of Genetics and Germplasm Innovation of Tropical Special Forest Trees and Ornamental Plants, Ministry of Education/Hainan Key Laboratory for Biology of Tropical Ornamental Plant Germplasm, College of Forestry, Hainan University, Haikou, 570228 China; 3 Manchester Institute of Biotechnology, University of Manchester, 131 Princess Street, Manchester, M1 7DN UK; 4 Present Address: Department of Biochemistry and Synthetic Metabolism, Max Planck Institute for Terrestrial Microbiology, 35043 Marburg, Germany; 5 State Key Laboratory of Ophthalmology, Zhongshan Ophthalmic Center, Sun Yat-sen University, Guangzhou, 510060 China

**Keywords:** Minimal genome, SCRaMbLE-based genome compaction, Essential gene array

## Abstract

**Background:**

Redundancy is a common feature of genomes, presumably to ensure robust growth under different and changing conditions. Genome compaction, removing sequences nonessential for given conditions, provides a novel way to understand the core principles of life. The synthetic chromosome rearrangement and modification by loxP-mediated evolution (SCRaMbLE) system is a unique feature implanted in the synthetic yeast genome (Sc2.0), which is proposed as an effective tool for genome minimization. As the Sc2.0 project is nearing its completion, we have begun to explore the application of the SCRaMbLE system in genome compaction.

**Results:**

We develop a method termed SCRaMbLE-based genome compaction (SGC) and demonstrate that a synthetic chromosome arm (synXIIL) can be efficiently reduced. The pre-introduced episomal essential gene array significantly enhances the compacting ability of SGC, not only by enabling the deletion of nonessential genes located in essential gene containing loxPsym units but also by allowing more chromosomal sequences to be removed in a single SGC process. Further compaction is achieved through iterative SGC, revealing that at least 39 out of 65 nonessential genes in synXIIL can be removed collectively without affecting cell viability at 30 °C in rich medium. Approximately 40% of the synthetic sequence, encoding 28 genes, is found to be dispensable for cell growth at 30 °C in rich medium and several genes whose functions are needed under specified conditions are identified.

**Conclusions:**

We develop iterative SGC with the aid of eArray as a generic yet effective tool to compact the synthetic yeast genome.

**Supplementary Information:**

The online version contains supplementary material available at 10.1186/s13059-020-02232-8.

## Introduction

A genome encodes both core functions that are essential for viability and accessory functions conferring better adaptation to different and changing niches. Through eliminating sequences that are nonessential for growth under optimal conditions in the laboratory, genome compaction studies not only greatly facilitate our understanding of the core principles of life, but also have the potential to create workhorses for biotechnology [1, 2]. The first minimal gene set (256 genes) was proposed in 1996 by comparing the first two available genomes, *Mycoplasma genitalium* genome and *Haemophilus influenzae* genome [3]. Since then, a series of theoretical and technical obstacles in gene essentiality evaluation and artificial genome synthesis have been solved by J. Craig Venter and colleagues across 20 years of creative works [4–7]. These led to the generation of the 531 kbp JCVI-syn3.0 genome (473 genes), a synthetic genome smaller than any known in nature capable of supporting independent growth [8]. Remarkably, 149 of the retained 473 genes are of unknown function, suggesting the presence of undiscovered core functions within prokaryotic life.

Compared to their prokaryotic counterparts, little is known about minimal eukaryotic genomes. Compacting the eukaryotic *Saccharomyces cerevisiae* genome is of great interest due to the wide application of yeast in both scientific research and industrial fermentation, but many challenges exist. The *S*. *cerevisiae* genome is 12 Mbp in length and composed of about 6000 genes [9]. This is much larger than the prototypical genome of JCVI-syn3.0, i.e., the 1 Mbp JCVI-syn1.0 genome (901 genes) [7]. This makes the “Design-Build-Test” cycle [8] or the sequential deletion [10], which was successfully used in prokaryotic genome compaction, time consuming and technically challenging. Furthermore, while gene deletions imply a greatly compacted genome could support yeast viability (about 80% of yeast genes were found to be nonessential in rich medium at 30 °C and 85% of these nonessential genes can be deleted individually while maintaining > 90% of the wild type growth rate in the same conditions [11, 12]), recent studies have shown that 90% of yeast genes have interactions and removing many of them results in synthetic lethality [13–15]. The synthetic lethality phenomenon indicates that a set of nonessential genes must be included alongside essential genes to sustain yeast growth. Unfortunately, given the complexity of genetic interactions and the context-dependent nature of gene essentiality [14, 15], it is almost impossible to nominate this set of nonessential genes for the design of minimal yeast genome(s).

Over the past decade, we have been working on the synthesis of world’s first synthetic yeast genome (Sc2.0), with six chromosomes having been successfully constructed [16–23]. One unique feature of the synthetic yeast genome is the genome-wide incorporation of loxPsym sites. Termed SCRaMbLE (Synthetic Chromosome Rearrangement and Modification by LoxPsym-mediated Evolution), this enables a system of inducible genome instability upon Cre recombinase expression [24]. A collection of papers have demonstrated the ability of SCRaMbLE to generate combinatorial genomic diversity via inversion, deletion, duplication, and translocation, in addition to the system’s diverse applications in metabolic engineering and strain evolution [25–35]. Given the deletion capacity of SCRaMbLE, it has been suggested to be an effective genome minimization tool [2, 24]. However, rearrangements generated by SCRaMbLE are quite random. Furthermore, the presence of essential genes prohibits some deletion events [30]. With the Sc2.0 project nearing completion, the establishment of an efficient workflow to maximize the deletion capacity of SCRaMbLE, with the final goal of generating minimal synthetic eukaryotic genomes, would be highly valuable.

## Results

### A SCRaMbLE-based method to efficiently compact synthetic chromosome

The embedded SCRaMbLE system in Sc2.0 provides a powerful tool to generate genotypic diversity by introducing chromosomal rearrangement such as deletion, inversion, duplication, and translocation. Among them, deletion leads to the loss of segments of DNA and therefore can be adopted to promote genome compaction. However, previous reports have shown that the rearrangements generated by SCRaMbLE are random and not all SCRaMbLEd strains have deletion events [28, 30–32, 36]. Thus, a method facilitating the rapid identification of SCRaMbLEd strains with deletion events will be helpful for utilizing SCRaMbLE as an efficient genome compaction tool.

**Fig. 1:**
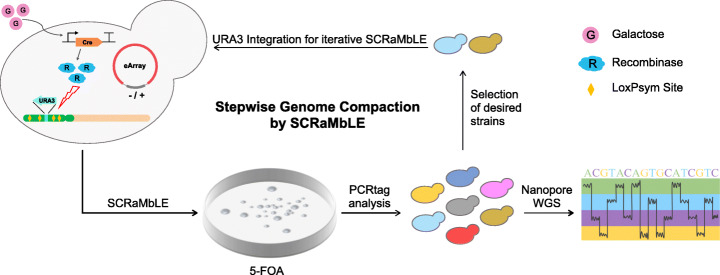
Schematic illustration of the SCRaMbLE-based genome compaction (SGC) method. The first step in SGC is to integrate the URA3 reporter into a nonessential LU. After SCRaMbLE, the cells are plated onto 5-FOA medium to select strains missing the URA3 reporter, within which rearrangements at other loci are highly likely to exist, as we reported previously [30]. The genomes of 5-FOA resistant strains are analyzed using PCRtags, followed by whole genome sequencing to identify sequence changes. Strains with the most synthetic sequences deleted are subjected to the next round of SGC

Given the widespread application of 5-FOA for the selection of Ura- cells [37], we proposed a SCRaMbLE-based genome compaction (SGC) method here. In this method, the URA3 reporter is integrated into the synthetic chromosome. 5-FOA is then used to select strains that have lost regions flanking the URA3 reporter during SCRaMbLE (Fig. 1). We tested this method using a yeast strain containing the synthetic left arm of chromosome XII (synXIIL) from our previous work [23]. SynXIIL contains 170 kbp of synthetic sequence (Additional file 1: Fig. S1) while the remaining right arm of chromosome XII is wild type. In total, 81 genes are encoded in the synthetic sequence, including 16 essential and 65 nonessential genes (Additional file 2: Table S1). Since SCRaMbLE in principle occurs only between loxPsym sites [25, 30, 38], we defined the sequence flanked by two adjacent loxPsym sites as one loxPsym unit (LU) for the convenience of subsequent description. SynXIIL is divided into 46 LUs, of which 9 contain at least one essential gene and LU-37 contains the centromere (Additional file 1: Fig. S2 and Additional file 2: Table S1). Zero to 8 genes are encoded in each unit with sequence lengths ranging from 50 bp to 16,404 bp.

The URA3 reporter was used to replace the YLL054C gene in LU-8 or the SSA2 gene in LU-24, as no growth defect was observed when these two genes were disrupted individually during the synthetic chromosome construction process and no essential genes are found in either LU [23]. In this study, a new Cre construct was used, instead of the daughter cell specific construct (pSCW11-Cre-EBD) used in previous study, with the aim of expanding Cre activity to all cells (Additional file 1: Fig. S3, the plasmid sequence is provided in Additional file 3: Table S2). A SV40 nuclear localization sequence (SV40-NLS) was added to the N-terminus of Cre recombinase to mediate nuclear localization and a GALS promoter was used to drive the expression of the SV40-NLS-Cre fusion protein. A detailed method to induce SCRaMbLE using this construct is provided in the “Methods” section.

We first induced SCRaMbLE in the synXIIL strain with URA3 reporter integrated in LU-8. Five 5-FOA resistant strains were randomly isolated and subjected to PCRtag analysis and subsequent nanopore sequencing. Found genome-wide, PCRTags are short pairs of primers designed in the Sc2.0 project by synonymous recoding. Expected amplicons should be detected using PCRtags if corresponding LUs are present (Additional file 1: Fig. S4). While PCRtag analysis provides a rapid view of the deletion landscape, nanopore sequencing is also required to assemble the compacted chromosome since other rearrangements besides deletion are present in SCRaMbLEd genomes and some loxPsym units are without PCRtag.

**Fig. 2:**
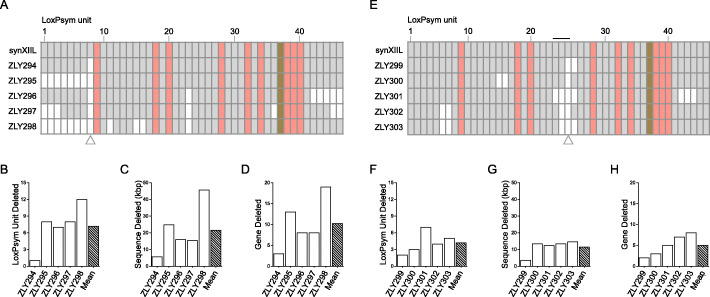
Compacting the left arm of synthetic chromosome XII (synXIIL) by SGC method. **a** The LU profiles of selected 5-FOA resistant strains after SCRaMbLEing the synXIIL strain with URA3 reporter in LU-8 (ZLY292). Nonessential LUs are shown in gray, essential-gene-containing LUs are shown in red, and centromere-containing LU are shown in brown. Deleted LUs are shown in white. The position of URA3 integration is marked by a hollow triangle. **b** The numbers of LUs deleted in strains in **a**. The average number of deleted LUs is also shown. **c** The lengths of deleted sequences in strains in **a**. The average length of deleted sequences is also shown. **d** The numbers of deleted genes in strains in **a**. The average number of deleted genes is also shown. **e**–**h** The LU profiles of selected 5-FOA strains after SCRaMbLEing the synXIIL strain with the URA3 reporter in LU-24 (ZLY293). The URA3 reporter was integrated at indicated sites by transforming the last chunks of megachunk A (for LU-8) or megachunk C (for LU-24), the fragments we used in our previous publication [23]. The integration of the last chunk of megachunk C introduces two additional loxPsym sites into LU-24 and divides LU-24 into three small LUs, 24-a, 24-b, and 24-c, which are indicated by the black line

Excluding ZLY294, in which only the LU-8 is deleted (Fig. 2a), the four other strains assessed all lost 7 or more additional LUs (Fig. 2b). On average, about 21 kbp of DNA sequences (Fig. 2c), corresponding to 10 genes (Fig. 2d), were deleted per strain. Particularly, a total of 12 LUs, spanning 45 kbp of synthetic sequences (over a quarter of synXIIL), were deleted in ZLY298 (Fig. 2a–d). Similarly, when the experiment was repeated in the strain with URA3 reporter integration in LU-24, the five selected 5-FOA resistant strains all lost more than one LU (Fig. 2f). The total deleted sequences ranged from 3 kbp to 14.5 kbp in length (Fig. 2g) and each strain had more than 2 genes deleted (Fig. 2h). Notably, the deleted LUs in all strains, except for ZLY294 and ZLY299, were not concatenated, suggesting at least two deletion events have occurred in these strains (Fig. 2a, e). Since previous results have shown the high stability of synthetic chromosome [17] and no colonies were identified by directly plating 2 × 10^4^ cells of ZLY292 or ZLY293 without Cre recombinase onto 5-FOA plates (Additional file 1: Fig. S5), we considered the deletions in ZLY294 and ZLY299 as the results of Cre-mediated deletion but not the results of spontaneous intra-chromosomal recombination, although this possibility cannot be excluded. Besides deletion, other rearrangements are also observed in these strains, the majority of which were inversions (Additional file 1: Fig. S6 and S7).

The requirement for positive selection using URA3 as a marker was further validated by comparing the deletion events can be identified by PCRtag analysis after one SCRaMbLE induction using synXIIL and ZLY293 (Additional file 1: Fig. S8). These two strains were SCRaMbLEd in the same way before synXIIL was plated onto SC medium and ZLY293 was plated onto SC + 5-FOA medium. Ten colonies on each plate were subjected to PCRtag analysis. Consistent with our previous SCRaMbLE results using synXII without any selection [31], no PCRtag deletions were identified in the 10 SCRaMbLEd synXIIL colonies. Conversely, 1 to 6 deleted loxPsym units were identified after SCRaMbLEing ZLY293 and selecting on 5-FOA, demonstrating the necessity of our SGC method.

As all the strains we identified after SGC harbor reduced synthetic chromosomes, notably with over a quarter of the synthetic sequence deleted in ZLY298, we concluded that our SGC method could offer an efficient tool to compact the synthetic yeast chromosome.

### One-step assembly of a compensatory essential gene array

LoxPsym sites are inserted 3 bp downstream of the stop codon of non-essential genes in the synthetic genome [16]. As a result, many essential-gene-containing LUs have additional nonessential genes within them (8 out of 9 in the case of synXIIL, Additional file 2: Table S1). Deleting any essential gene in these LUs results in an inviable strain [11, 12]. Consequently, none of the 9 essential-gene-containing LUs were deleted by SGC, as shown in Fig. 2. The nonessential genes located in these LUs could not be removed by SGC directly and thus limit the compacting ability of SGC. One way to overcome this problem is to introduce an episomal essential gene array which could functionally compensate the loss of native essential genes.

**Fig. 3:**
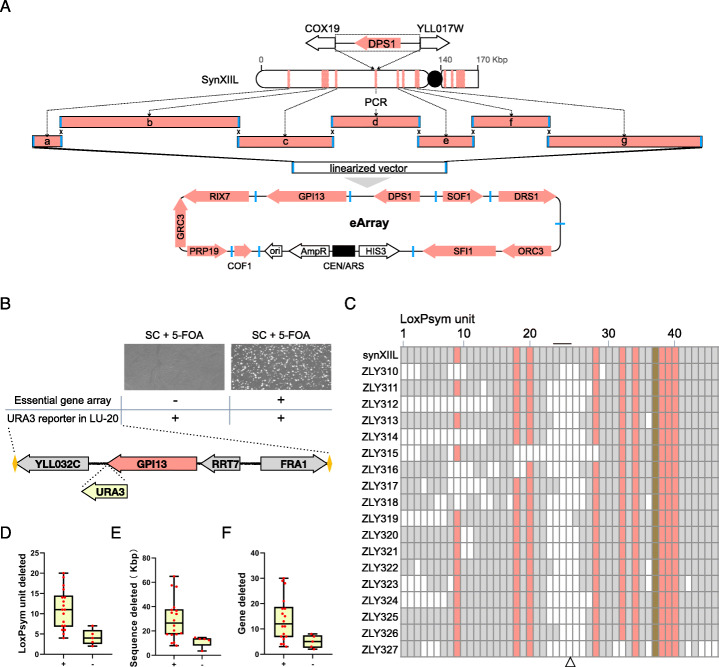
The presence of eArray expands the compacting ability of SGC. **a** The design and content of eArray. The 10 essential genes assembled in eArray distribute across 7 loci (a–g) in the first 140 kbp of synXIIL. Locus d is shown in detail with sequences that were copied from synXIIL to eArray. Other loci are defined similarly, except for the left end of locus b, in which only 110 bp are used to avoid the inclusion of complete YLL037W open reading frame. Homologous sequences (shown in blue) were added to these 7 loci by PCR. PCR products and a linearized vector were co-transformed into yeast to assemble eArray. The genes encoded and other functional elements in the eArray are shown at the bottom. The centromere-containing sequences are shown in black. **b** Plating results of SGC in strains with or without eArray. The URA3 reporter was integrated between the essential gene GPI13 and nonessential gene YLL032C in LU-20 in these two strains before SGC. Equal amount of cells were plated after SGC. **c** The LU profiles of selected 5-FOA resistant strains after SGC. ZLY307 with the URA3 reporter in LU-24 and eArray was SCRaMbLEd and plated onto 5-FOA plates. This figure uses the same design strategy as Fig. 2a. **d** A box plot showing the distribution of number of LUs deleted in each strain in the group with eArray (+) in **b** and the group without eArray (−) in Fig. 2e. **e** A box plot showing the distribution of lengths of deleted sequences in each strain in the group with eArray (+) in **b** and the group without eArray (−) in Fig. 2e. **f** A box plot showing the distribution of numbers of genes deleted in each strain in the group with eArray (+) in **b** and the group without eArray (−) in Fig. 2e

An essential gene array (denoted as eArray hereafter) was designed to test this possibility (Fig. 3a, eArray sequence is provided in Additional file 3: Table S2). In order to compare the compacting ability of SGC with or without additional copies of essential genes, only the 10 essential genes from the first 140 kbp of synXIIL were included in the eArray. The remaining 6 essential genes in the rest 30 kbp of synXIIL were not included and used as internal controls (Fig. 3a). These 10 essential genes were located across 7 loci (locus a to g in Fig. 3a), with locus b containing three essential genes (PRP19, GRC3 and RIX7) and locus g containing two (SFI1 and ORC3). For each locus, the chromosomal sequences flanked by the two nearby nonessential coding sequences were used in eArray. This facilitated the retention of the native regulation of essential genes while minimizing nonessential coding sequences. PCR primers were designed to amplify these loci and add overhangs of at least 40 bp to direct the ordered assembly of a centromeric plasmid that could propagate in yeast cells and be selected by SC-His medium (Fig. 3a). The relative position and direction of essential genes were not changed and no loxPsym sites were included in the eArray to ensure its stability during SGC.

We constructed the eArray in yeast by utilizing its high recombination efficiency. Essential genes were amplified using synXIIL genomic DNA as template and transformed together with a linearized pRS413 vector into the wild type BY4742 strain (Additional file 1: Fig. S9A). His+ colonies were screened by PCRtag analysis to confirm the presence of transformed sequences (Additional file 1: Fig. S9B). Junction PCR was used to verify the assembly of these sequences in the designed order (Additional file 1: Fig. S9C). Seven out of the 8 randomly picked were positive for all PCRtags and junctions. One of these 7 colonies was used for further eArray recovery. Three restriction analyses were performed using the recovered eArray and all produced the predicted results (Additional file 1: Fig. S9D-F), suggesting the eArray was correctly constructed.

To confirm the functionality of essential genes in the eArray, we utilized a tetrad analysis assay. After introducing the eArray into individual heterozygous strains, each missing one genomic copy of one of the eArray-based essential genes [11, 12], we found that for all 10 essential genes the presence of the eArray could support viability of all four spores on the YPD medium, as shown in Additional file 1: Fig. S9G. This indicated all corresponding genes within eArray were functional. In addition, as indicated by the similar sizes of the four spores (Additional file 1: Fig. S9G) and comparable growth with BY4742 (Additional file 1: Fig. S9H), the relocation of genes onto the eArray the showed little impact on cellular fitness under the condition tested.

These results suggested that clustering the essential genes into one array is feasible and can compensate for the loss of chromosomal essential genes nicely.

### The presence of the eArray enhances the compacting ability of SGC

We first tested whether the presence of the eArray could facilitate the deletion of essential-gene-containing LUs by integrating a URA3 reporter at LU-20, an LU that contains one essential gene (GPI13) and three nonessential genes (YLL032C, RRT7 and FRA1). In contrast to the strain without the eArray, where no clones are able to survive on the SC medium containing 5-FOA, many colonies appeared after SGC when the eArray was present, indicating the success of LU-20 deletion (Fig. 3b). Three of these colonies were picked for PCRtag analysis, revealing the loss of LU-20 specific PCRtags (Additional file 1: Fig. S10). These results demonstrated that the presence of the eArray could facilitate the deletion of LU-20 by SGC; moreover, the three nonessential genes within LU-20 can be deleted simultaneously without affecting yeast cell viability.

We then tested the effect of the eArray on the compacting ability of SGC using ZLY293, a strain housing the URA3 reporter within LU-24. This strain was chosen because of its relatively limited capacity for SGC, as shown in Fig. 2e. After transforming the eArray into this strain, SGC was performed and 18 5-FOA-resistant colonies (ZLY310-327) were analyzed by nanopore sequencing.

We found that 5 out of the 9 essential-gene-containing LUs (colored by red) were deleted by SGC across different strains, suggesting the presence of the eArray expands the deletion activity of SGC to these LUs by decoupling essential and nonessential genes (deletion profiles of these 18 strains are shown in Fig. 3c). Furthermore, this result also suggests that the nonessential genes within these LUs are dispensable together with other nonessential genes deleted in these strains. Among the remaining 4 essential-gene-containing LUs, preservation of LU-38, LU-39, and LU-40 was expected as they were used as internal controls and the essential genes in these LUs were not included in the eArray. LU-34 contains two quasi-essential genes MMM1 and RTT109, which both cause significant growth defects once deleted [23, 39, 40].

We compared the number of deleted LUs, the size of deleted sequences, and the number of deleted genes after SGC in strains with (+) or without (−) the eArray (Fig. 3d–f). The maximum values of these three parameters in the (+) group were 20 LUs (total 46 LUs), 64 kbp (total 170 kbp) and 30 genes (total 81 genes), *i.e.*, more than one third of the synthetic sequence was deleted through only one round of SGC with the aid of the eArray. The corresponding maximum values in the (−) group were 7 LUs, 14 kbp, and 8 genes. Compared to the (−) group, the mean value of these three parameters in the (+) group increased from 4 LUs to 11 LUs, 11 kbp to 30 kbp, and 5 genes to 14 genes. The minimum values also increased in the (+) group compared to the ones in the (−) group, from 2 LUs to 4 LUs, 3 kbp to 7 kbp, and 2 genes to 3 genes, though the increase was not that obvious compared to the maximum and mean values. The wider distribution range of these three parameters suggests that more deletion events are permitted in the presence of the eArray. More quantitatively, the compacting ability of SGC is nearly tripled for these three parameters in the presence of the eArray.

With these data, we concluded that, in the presence of the eArray, the compacting ability of SGC is enhanced not only by enabling the deletion of nonessential genes located in the essential-gene-containing LUs, but also by allowing more chromosomal sequences to be deleted in a single SGC process.

### The deletion boundary reflects the 3D proximity of loxPsym sites

Although the presence of the eArray expands general compacting ability, the deleted LUs flanked LU-24 are mostly restricted from LU-23 to LU-27 (Fig. 3c). This cannot be a result of the synthetic lethal effect, as the sequence from LU-18 to LU-28 is deleted in strain ZLY315 by SGC (Fig. 3c) and the sequences from LU-21 to LU-27 could be deleted using the CRISPR/Cas9 method (Additional file 1: Fig. S11A).

Cre-loxP-mediated deletion requires the formation of a synapsed structure by four Cre recombinases, pairs of which recognize a loxPsym site [41]. If two loxPsym sites (for example, the a and b loxPsym sites in Additional file 1: Fig. S11B) are located in the same chromosomal interacting domain (CID), the Cre recombinases binding them are more likely to come into contact with each other to mediate tetramer formation due to their 3D proximity, compared to two loxPsym sites located in different CIDs (for example, the a and c loxPsym sites in Additional file 1: Fig. S11B). Therefore, the closer the two loxPsym sites are within the 3D genomic structure, the more likely they are to mediate deletion events. Similarly, the restricted deletion range we observed here may reflect the 3D proximity of loxPsym sites from LU-23 to LU-27. To test this, we extracted and analyzed the 1-170 kbp Hi-C data of synXII from our previous publication [42]. The sequence from LU-23 to LU-27 perfectly matches a CID detected by our Hi-C analysis (labeled by an asterisk in Additional file 1: Fig. S11C). This is to say the loxPsym sites outside LU-23 to LU-27 are in different CIDs; thus, they are hard to mediate URA3 deletion.

A previous work in mice also showed results similar to us, suggesting that Cre recombination efficiency is inversely proportional to the genetic distance between *loxP* sites [43]. Given this, we next asked whether this is applicable to other regions in synXIIL. Since the strains in Fig. 3c were exposed to Cre activity for only 2 h, we considered deleted, concatenated LU the result of single deletion event. Thus, according to our hypothesis, most deletion events we detected in these strains should have sequence lengths equal to or shorter than the lengths of CIDs. The size distribution of deleted segments in these strains were calculated and shown in Additional file 1: Fig. S11D; the relative short deletion length (mean = 10 kbp, medium = 7 kbp) is comparable to the limited intra-chromosomal interaction range of synXIIL (about 10 kbp at each side, Additional file 1: Fig. S11E), which is also consistent with the Micro-C results reported by Hsieh and colleagues [44] that CIDs in yeast are typically 2–10 kbp in length. These results further support our hypothesis that loxPsym sites located in the same CID more favorably mediate recombination due to their 3D proximity.

In summary, a positive correlation between the proximity of loxPsym sites in 3D structure and the likelihood of these loxPsym sites meditating a deletion event is revealed by our data, suggesting the 3D contact information of loxPsym sites can potentially be used to guide the selection of URA3 integration sites. It will be of interest to test this observation in more complex situations, for example, in a multi-synthetic chromosome background, to see whether it will be easier to delete more LUs in a single SGC process if URA3 is integrated into a CID covering longer sequence.

### Stepwise chromosome compaction by iterative SGC

Since SCRaMbLE is random and only limited numbers of clones can be analyzed in detail, it will be difficult, if not impossible, to obtain strains with maximally compacted chromosome through one round of SGC. Stepwise chromosome compaction by iterative SGC may be a possible solution to this. Thus, we explored this possibility here.

**Fig. 4:**
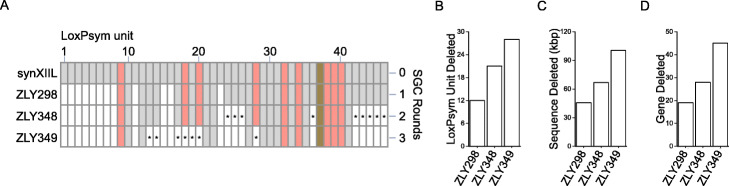
Stepwise chromosome compaction by iterative SGC. **a** Stepwise chromosome compaction by three rounds of iterative SGC. The LU profiles of selected 5-FOA resistant strains after SGC are shown using the strategy as detailed in Fig. 2a. The newly deleted LUs in each round of SGC are indicated by asterisks. **b** The number of LUs deleted in strains in **a**. ZLY298 is identified after the first round of SCRaMbLE. ZLY348 is from the second round of SGC and ZLY349 is from the third round of SGC. **c** The lengths of deleted synthetic sequence in strains in **a**. **d** The numbers of deleted genes in strains in **a**

Among the 10 strains from the initial SGC experiment, without the eArray in Fig. 2, ZLY298 has the maximal chromosome reduction (~ 45 kbp/170 kbp, 26% of synXIIL sequences removed, Fig. 2c) and therefore was chosen as the target for further compaction. A URA3 reporter was integrated into LU-24 prior to a second round of SGC induction. PCRtag analysis was performed to screen 12 randomly isolated 5-FOA-resistant strains. This identified ZLY348 as the candidate strain with the most additional deleted LUs. This strain was subjected to nanopore sequencing. The sequencing results showed that 9 additional LUs were deleted (Fig. 4a, b), resulting in a strain with 41% of synXIIL sequence (~ 71 kbp/170 kbp) being removed (Fig. 4c). The total length of the synthetic sequences in ZLY348 is 99 kbp, including 25 of the original 46 LUs (Fig. 4b, c). All 10 essential LUs are maintained in ZLY348 (Fig. 4a).

Next, the eArray was transferred into ZLY348, with a URA3 reporter inserted into LU-20 before the third round of SGC. After SGC induction, PCRtag analysis was performed among 10 randomly isolated 5-FOA-resistant clones. This identified ZLY349 as the candidate strain with the most additional deleted LUs. Sequencing data showed that 7 additional LUs were deleted (Fig. 4b), including 3 LUs containing essential genes (Fig. 4a), resulting a strain with 58% of synXIIL sequence (~ 100 kbp/170 kbp) removed (Fig. 4c). Only 18 of the 46 LUs were retained in this strain. ZLY349 is the strain with the most synthetic sequence deleted in this study, as further attempts to compact the synthetic sequence in ZLY349 failed.

Although inversions are also present in these strains, step-wise reduction of the overall synthetic sequence length is seen during the iterative SGC process (Additional file 1: Fig. S12). We therefore propose the application of iterative SGC as an effective synthetic genome compaction method which could lead to a more thorough compaction of yeast genome.

### Trade-offs among genome size, growth rate, and growth condition

An organism’s minimal genome is situational, depending on its genomic context and external environment [45, 46]. About half of the genome of JCVI-syn1.0 was removed in JCVI-syn3.0, resulting in a 3-fold decrease of growth rate in rich medium [8]. The trade-offs among genome size, growth rate, and growth condition should be handled carefully during genome compaction.

**Fig. 5:**
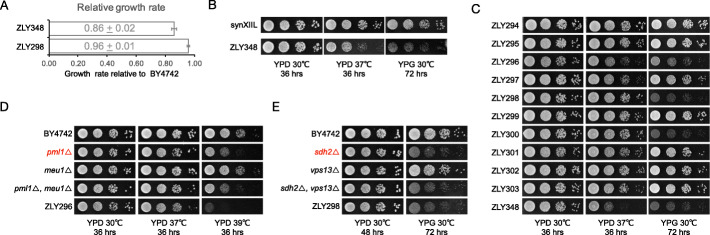
Trade-offs among genome size, growth rate, and growth condition. **a** Growth rates relative to the wild type strain BY4742. The growth curves of ZLY348, ZLY298, and BY4742 were measured and maximum growth rates were calculated as we described previously [30]. The growth rate of BY4742 was normalized to 1 and the growth rates of ZLY348 and ZLY298 were shown as mean ± SD. Six replicates for each strain were used to calculate the mean and standard deviation. **b** Tenfold serial dilution was performed for ZLY348 and synXIIL under indicated conditions, and plates were imaged at the indicated time after plating. **c** Tenfold serial dilution was performed for indicated strains under conditions identical to **b** and plates were imaged at the indicated time after plating. **d** PML1, but not MEU1, is required for high temperature tolerance. Tenfold serial dilution was performed for these strains onto the indicated medium, and plates were imaged at the indicated time after plating. **e** SDH2, but not VPS13, is required for the utilization of glycerol as carbon source. Tenfold serial dilution was performed for these strains onto the indicated medium, and plates were imaged at the indicated time after plating

We first monitored the growth of all sequenced strains generated in this study on solid YPD medium at 30 °C, an optimal growth condition in the laboratory [47]. As shown in Additional file 1: Fig. S13, despite the loss of various chromosomal sequences, 30 out of the 40 sequenced strains showed similar growth to that of wild type under this condition. Among these 30 strains, ZLY298 and ZLY348 had the most synthetic sequence being deleted. Their growth rates in liquid YPD medium at 30 °C were therefore measured to give a more quantitative description. As shown in Fig. 5a, although no obvious growth defects can be observed on solid YPD medium, the growth rate of ZLY348 was slightly below the previously used 90% cutoff [11, 12]. Alternatively, ZLY298 has a growth rate almost the same as that of the wild type. These data suggested that the 18 genes deleted in ZLY298 (Additional file 4: Table S3) are not required for robust growth in rich medium at 30 °C and the deletion of the 9 additional genes in ZLY348 (Additional file 4: Table S3) causes a slight decrease of growth rate in liquid YPD medium. If a more severe growth defect is tolerated, 39 out of the total 65 nonessential genes in synXIIL can be deleted (ZLY349 in Additional file 1: Fig. S13 and Additional file 4: Table S3).

The preservation of genes not required for robust growth in the wild type genome suggests that they may have functions that are needed under non-optimal conditions. We tested this hypothesis by comparing the growth of ZLY348 and synXIIL under conditions including high temperature (37 °C) and non-fermentable carbon source (YPG). Growth defects were observed for ZLY348 under these conditions (Fig. 5b), indicating the loss function of related genes. Similar growth defects were also observed in strains with different compacted synXIILs (Fig. 5c). The common deletions shared by these strains but not present in the remained normal strains will be the possible causal factors for corresponding defects. For example, LU-45 and LU-46 are such common deletions shared by strains showing growth defects at 37 °C (ZLY296, ZLY297, and ZLY348 in Fig. 2a, 4a, and 5c). The deletion of LU-45 and LU-46 results in the loss of two genes, PML1 and MEU1 (Additional file 2: Table S1). The deletion of PML1 was previously showed to cause high temperature sensitivity [48], but it is not clear whether the function of MEU1 is related to this. Further deletion of these two genes in wild type strain individually or combinatorially revealed PML1 but not MEU1 as a functional gene required for high temperature tolerance (Fig. 5d). It is worth mentioning that the high temperature sensitivity induced by *pml1* deletion is not as severe as that seen in ZLY296 or ZLY348, suggesting other factors in these strains also contribute to their growth defects. Similarly, we identified SDH2 in LU-15 but not VPS13 in LU-16 as a functional gene for growth on YPG medium (Fig. 5e).

Our data suggested that, in a given condition, the extent to which the yeast genome can be compacted depends on the minimum acceptable growth rate. With the difficulty of pinpointing an acceptable growth rate and contextual parameters required for a minimal genome, we propose the generation of minimal genomes that can support the robust growth in YPD medium at 30 °C as an executable and achievable goal in the future. This will further contribute to understanding and designing environmental adaptivity within the yeast genome by evaluating the functions of deleted genes in non-optimal conditions.

## Discussion

The compaction of model genomes provides a way to characterize the core biological networks that are key to understand the laws and origins of life. Organisms with compacted genomes may be better factories due to the effective use of intracellular material and energy resulting from the elimination of unnecessary processes. Top-down or bottom-up genome compaction methods have been applied to organisms including *Escherichia coli* [10], *Bacillus subtilis* [49, 50], *Mycoplasma mycoides* [8], *Saccharomyces cerevisiae* [51], and *Schizosaccharomyces pombe* [52]. Compared to the reported 5% reduction in *S*. *cerevisiae* genome by chromosome-splitting and losing techniques [51], the designed synthetic yeast genome is smaller than the natural one in size by nearly 8%, due to the deletion of sequences including retrotransposons, introns and LTR repeats [19]. Additionally, the SCRaMbLE system implemented in the synthetic genome makes it possible to generate variants with further compacted genomes [24]. The SCRaMbLE system is unique to any other reported genome compaction method as it has no requirement for pre-evaluation of synthetic lethality and has the ability to generate varied compacted genomes by reshuffling LU segments. Several aspects of SCRaMbLE-based genome compaction (SGC) method are addressed in this paper, paving the way for its use as an effective synthetic yeast genome compaction tool:

First, not all genomes contain deletions after SCRaMbLE. URA3 integration and 5-FOA selection is used in the SGC method to quickly identify strains containing at least one deletion event and enrich the population for reduced genome sizes. Since the URA3-integrated LU will be excluded in the selected genomes, careful experimental design should be applied to its location. In this study, we chose LUs that do not affect robust growth once deleted. Our data also suggested the LUs located in CIDs covering longer sequences will also be good choices. The possible choice of URA3 insertion site will increase exponentially with the number of synthetic chromosomes. Any one of the possible URA3 integration sites can be used in the first round SGC and the sites retained after this round of SGC can be used in the next round. Several integration sites can be tested in parallel in one SGC process. The phenomenon that no colonies can be obtained or all colonies are smaller than their parent after SGC indicates genes in the URA3-integrated LU are required in minimal genomes. Since gene essentiality is context dependent, it is worth further testing whether the choice of different sites for URA3 integration would result in different minimal genomes [45].

Second, the drawback of SGC is that a URA3-integrated LU will be excluded in the compacted genome. Because the number of rearranged genomes is extremely large after SCRaMbLE, to identify all compacted genomes is a nearly impossible goal using currently known methods. Different compacted genomes may produce a similar biological fitness under defined growth conditions and our method can generate some of them, as we have shown in Fig. 5 and Additional file 1: Fig. S13. Without URA3 integration and 5-FOA selection, PCRtag analysis can be used to identify strains with deletions if enough colonies are screened. By using this marker-free method, the locations of the deletion events would be less restricted and less biased. However, given that thousands of PCRtags will present in the final Sc2.0 strain, it will be highly labor-intensive to identify strains with reduced genomes for use in further rounds of SGC. Combining SCRaMbLE with lab adaptive evolution will increase the chances of deletion events over many generations; enough SCRaMbLE and evolution time will theoretically result in clones with at least one deletion. Methods that can accurately, sensitively, and quickly measure the DNA content in a single cell are needed to screen SCRaMbLEd cells with deletions.

Third, genes located in the same LU will be retained or removed together; thus, the nonessential gene(s) located in essential gene(s) containing LUs cannot be eliminated by SCRaMbLE directly. The presence of the eArray expands the compacting ability of SGC by getting rid of the content and range limits of SCRaMbLE mediated deletion. Clustering the essential genes into one array compensates for the loss of chromosomal essential genes nicely as suggested by the tetrad dissection results (Additional file 1: Fig. S9G) and serial dilution (Additional file 1: Fig. S9H), but a full recovery of regulation and function may not be assured. A co-culture assay was performed to directly compare the fitness of these strains with synXIIL and BY4742 (Additional file 1: Fig. S14). Although nearly equal number of cells were present at the start of co-culture, the percentage of eArray containing cells was only half that of synXIIL or BY4742 after 3 rounds of 24-h co-culture, indicating the competition ability of these eArray containing strains was lower than synXIIL or BY4742. This strategy can be applied for compacting the whole yeast genome by synthesizing an essential megachromosome containing all essential genes, although attention should be paid to the dose effect of essential genes during its construction [53–55]. Ideally, every gene should have the same possibility to be SCRaMbLEd individually to allow the dissection of the essentiality of individual genes. Our solution provides the first step in this direction by introducing an essential gene array. A more thorough solution could be the synthesis of a redesigned yeast genome that has only one gene in each LU, but additional efforts are required to design and construct such a genome.

Fourth, the extreme diversity of compacted genomes after SGC makes it difficult to obtain strains with maximally compacted chromosome through one round of SGC. Therefore, multiple rounds of SGC are applied. Combination of PCRtag analysis and long-read sequencing techniques, such as nanopore sequencing, provides a reliable method to efficiently identify strains with compacted genomes. PCRtag analysis provides an economical and rapid solution for pre-screening and whole genome sequencing can ultimately reveal the exact genome structure. The combination of these two methods is necessary as other rearrangements such as inversion and duplication will happen simultaneously in the compacted genomes and some LUs contain no PCRtags. The slow growth phenotype is not an effective indicator for a small genome, since it may simply highlight lost genes that are critical for cell growth and not necessarily more deleted sequences, for example, the ZLY295 strain. As we have shown both here and previously [23], duplication is rare in the SCRaMbLEd synXII or synXIIL genome. Duplications are not likely to largely affect the SGC process because they have the possibility to be deleted during subsequent rounds of SGC. Alternatively, they may be removed by integrating the URA3 reporter into the duplicated LU(s).

Finally, the extent to which the yeast genome can be compacted depends on the growth conditions and desired growth rate. The simplicity and effectiveness of the SGC process allow the selection and comparison of compacted genomes supporting different growth rates under different conditions, which can be used to clarify the core genomes for viability and the laws of genomic robustness. Ideal workhorses for biotechnology are the strains without unnecessary wastage of cellular energy and material, but with robust growth under the required condition. Thus, we did not insist on seeking for a genome smaller than the one of ZLY349 due to its significant growth defect. We propose the generation of minimal genomes that can support robust growth in YPD medium by iterative SGC with the aid of eArray as an executable and achievable goal in the future. This will provide valuable insights for the understanding and further design of the environmental adaptivity of yeast.

## Methods

### Medium, PCRtags, and strains used

Media used in this study was prepared as previously reported [23, 47]. Briefly, the YPD medium was made by mixing autoclaved 2 X YEP stock (40 g/L peptone, 20 g/L yeast extract, 40 g/L glucose, and 0.64 g/L tryptophan) with autoclaved 4% (w/v) agar in equal volume. 5-FOA media was made by dissolving 0.5 g 5-fluorouracil-6-carboxylic acid monohydrate into 250 mL autoclaved 2 X SC stock (4.25 g/L Difco yeast nitrogen base w/o amino acids, 12.5 g/L ammonium sulfate, 5 g/L amino acid drop-out mix), before mixing with an equal volume of autoclaved 4% (w/v) agar for plate preparation. The YPG plates were prepared using the same process as YPD plates but with 3% glycerol as a carbon source instead of glucose. PCRtags were used as per our previous publication [23]. The strains used in this study are listed in Additional file 5: Table S4.

### Yeast transformation

Lithium acetate (LiOAc) mediated yeast transformation was used as previously described [23]. Briefly, a single yeast colony was inoculated in 5 mL appropriate medium and cultured overnight at 30 °C with shaking at 220 rpm. Overnight culture was diluted to OD_600_ = 0.1 in 5 mL fresh medium and cultured at the same condition for 4–6 h until the OD_600_ reaches 0.4–0.6. Cells were collected by centrifugation at 3000 rpm for 5 min, washed with 1 mL sterile water and 1 mL 0.1 M LiOAc respectively, and then resuspended in 100 μL 0.1 M LiOAc. Transformation buffer (312 μL 50% PEG3350, 41 μL 1 M LiOAc and 25 μL ssDNA) was mixed with 100 μL competent cells and DNA fragments and/or plasmids, and incubated at 30 °C for 30 min. Fifty microliters of DMSO was then added into the mixture and distributed by gentle inversion before heat-shock for 15 min in a 42 °C water bath. Cells were pelleted, washed with 1 mL 5 mM CaCl_2_, and then plated onto selective agar medium.

### SCRaMbLE induction

A single yeast colony was inoculated into 5 mL selective medium with 2% w/v glucose and cultured overnight at 30 °C. 1/10 of the overnight culture was added into 5 mL of new medium with 2% w/v raffinose and 0.1% w/v glucose and grown for 4 h or more to mid-log (OD_600_~0.4). Galactose stock to 2% w/v final concentration was added and cultures were incubated at 30 °C for an additional 2 h to induce Cre expression. Cells were collected and washed with 1 mL sterile water once, resuspended in 50 μL sterile water, and plated onto 5-FOA medium.

### Nanopore sequencing and SCRaMbLEd genome assembly

High-quality DNA extraction was performed according to a modified Qiagen Genomic-tip 100/g protocol with the Qiagen Genomic Buffer kit. DNA quality was accessed via gel-electrophoresis, NanoDrop™ 2000 Spectrophotometer, and Qubit 4 Fluorometer using the dsDNA BR reagent. Library preparation was performed using the SQK-LSK108 library kit with the Native Barcoding kits EXP-NDB104 and EXP-NDB113 according to the manufacturing guidelines. The only alteration was to not shear the input DNA but increase the starting DNA concentration 5-fold to match the molarity expected in the protocol, based on our experience with PFGE analysis of similar prepared genomic DNA (data not shown). Sequencing was performed on a MinION Mk1B device using FLO-MIN106D with R9.4.1 chemistry. DNA Sequencing was performed for 48 h using MinKNOW (v19.05.0) software.

Base calling and demultiplexing was performed locally using Guppy (v3.1.5) software. The obtained data was mapped against the reference genome of BY4741+synXIIL using minimap2 (v2.17) [56] and NGMLR (v0.2.7) [57]. Rearrangements in the NGMLR mapping data were called using Sniffles (v1.0.11) [57] with a threshold of ≥ 10 reads confirming the rearrangement. De novo genome assembly of each strain was performed using Canu (v1.8) [58]. The evaluation and confirmation of SCRaMbLEd rearrangements in synXIIL was done by comparing the mapping and variant calling data from Sniffles with the de novo assembly obtained by Canu.

The raw datasets of ZLY292-309 from the ONT sequencing platform were first corrected by NECAT [59]. We used two overlapping-error-rate thresholds to select supporting reads after filtering via DDF scoring and k-mer chaining. The global overlapping-error-rate threshold was set to 0.5 and 200 candidate reads with top DDF scores were selected for local alignments. The 500 bp block size was used to align candidate reads to a template, and threshold value 12 was used to filter the matched candidate reads; we then used the FALCON-sense consensus algorithm and corrected error-prone nanopore reads in two steps by the progressive method. The 10% trimmed reads were then used for pairwise alignments and filtering low-quality read overlaps. The overlapping high-quality reads were assembled using the two-step progressive genome assembler as described in NECAT. To access the detailed positions of the 46 loxPsym units in the 18 de novo assemblies, we aligned the segments to each assembly and extracted the insertion and deletion regions using Nucmer [60]. The candidate SV regions and unmapped segments were all validated using Blast local alignment.

### Hi-C analysis of synXIIL

Raw synXII Hi-C data was downloaded from the NCBI SRA database (accession number: SRR3173861, [42]). In total, 3.2 Gbp of raw data was obtained. After filtering low quality reads, clean data was mapped to the synXII chromosome using BWA-MEM read alignment tools [61] using parameter -A1 -B4 -E50 -L0. Hi-C contact matrix was constructed by hicBuildMatrix in the HiCExplorer software package [62] with parameter--binSize 2000. hicCorrectMatrix was then used to correct the matrix data with parameter --filterThreshold -1.5 5. Finally, Hi-C contact matrix heat map was profiled by hicPlotMatrix —log 1p. We then generated an ICE-normalized contact matrix using this data by HiC-Pro [63]. The bowtie2 options were set to very-sensitive mode in HiC-Pro configuration file. Contacts on synXII with intensity higher than 5 in the previous contact matrix are illustrated in the figure S11.

## Supplementary Information

**Additional file 1: Fig. S1.** Map of synXIIL. **Fig. S2.** Gene number and size of each loxPsym unit of synXIIL. **Fig. S3.** Map of the Cre plasmid used in this study. **Fig. S4.** An example of PCRtag analysis. **Fig. S5.** Stability of URA3 integrated in synXIIL without the expression of Cre. **Fig. S6.** Genome rearrangements in ZLY294-ZLY298. **Fig. S7.** Genome rearrangements in ZLY299-ZLY303. **Fig. S8.** PCRtag analysis of SCRaMbLEd strains with or without URA3 integration and 5-FOA selection. **Fig. S9.** The assembly and verification of eArray. **Fig. S10.** PCRtag analysis to confirm the deletion of LU-20. **Fig. S11.** The deletion boundary reflects the 3D proximity of loxPsym sites. **Fig. S12.** Genome rearrangements in iterative SGC strains. **Fig. S13.** The growth of SGC strains on YPD 30 °C. **Fig. S14.** Competition among synXIIL, BY4742 and eArray strain with single essential gene deletion.

**Additional file 2: Table S1.** The information of loxPsym fragments in synXIIL.

**Additional file 3: Table S2.** The sequence of plasmids used in this study.

**Additional file 4: Table S3.** List of genes deleted in iterative SGC.

**Additional file 5: Table S4.** Strains used in this study.

**Additional file 6.** Review history.

## Data Availability

All data, plasmids, and strains used for this paper are available from the authors upon request. Raw synXII Hi-C data was downloaded from the NCBI SRA database with accession number: SRR3173861 [64]. The sequencing data of the SCRaMbLEd strains have been deposited into NCBI BioSample database with the BioProject accession number: PRJNA661458 [65].
