# Supplementary material for: Compacting a synthetic yeast chromosome arm
Source: Genome Biol. 2021 Jan 4;22:5. doi: 10.1186/s13059-020-02232-8 (PMC7780613; doi:10.1186/s13059-020-02232-8)
Supplement: Supplementary file 1 — Additional file 1: Fig. S1. Map of synXIIL. Fig. S2. Gene number and size of each loxPsym unit of synXIIL. Fig. S3. Map of the Cre plasmid used in this study. Fig. S4. An example of PCRtag analysis. Fig. S5. Stability of URA3 integrated in synXIIL without the expression of Cre. Fig. S6. Genome rearrangements in ZLY294-ZLY298. Fig. S7. Genome rearrangements in ZLY299-ZLY303. Fig. S8. PCRtag analysis of SCRaMbLEd strains with or without URA3 integration and 5-FOA selection. Fig. S9. The assembly and verification of eArray. Fig. S10. PCRtag analysis to confirm the deletion of LU-20. Fig. S11. The deletion boundary reflects the 3D proximity of loxPsym sites. Fig. S12. Genome rearrangements in iterative SGC strains. Fig. S13. The growth of SGC strains on YPD 30 °C. Fig. S14. Competition among synXIIL, BY4742 and eArray strain with single essential gene deletion. [file 13059_2020_2232_MOESM1_ESM.docx]

**Fig S1. Map of synXIIL
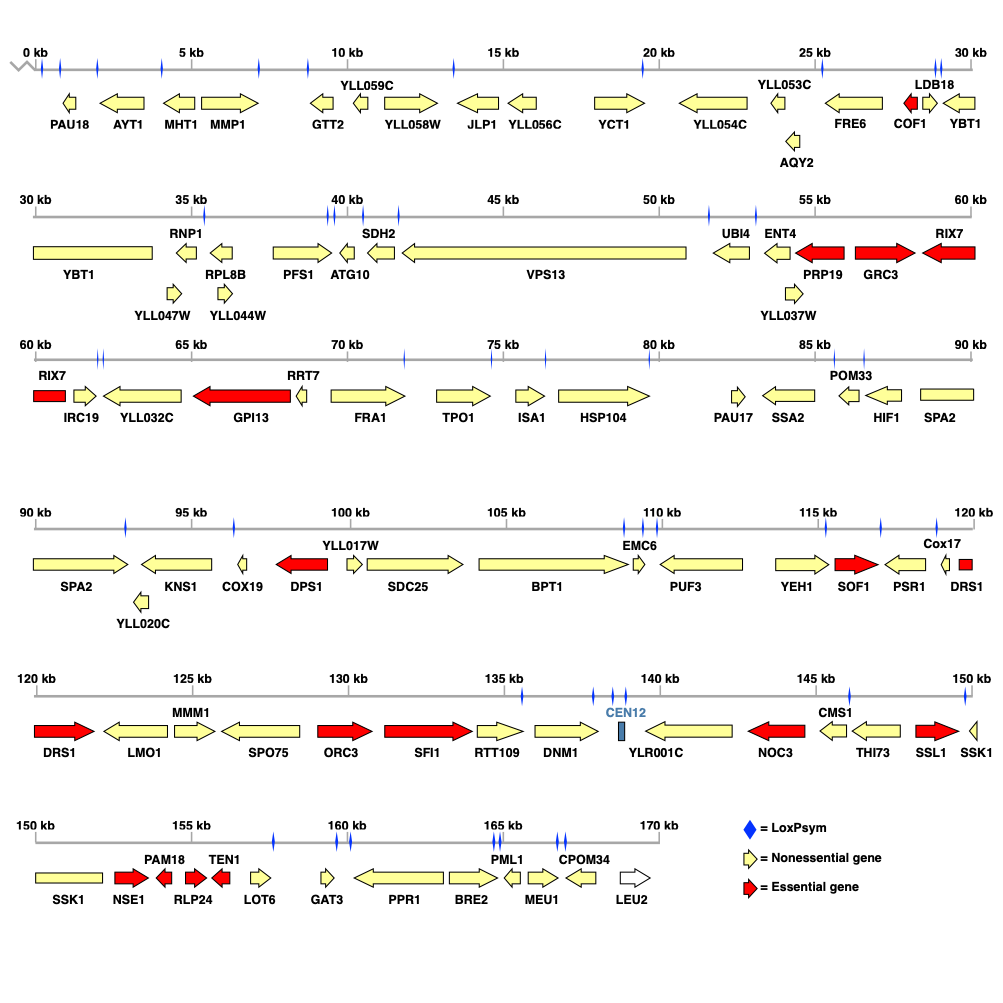
**

The synthetic sequence of synXIIL strains is shown with the encoded genes and loxPsym sites. Essential genes are shown in red.

**Fig S2. Gene number and size of each loxPsym unit of synXIIL
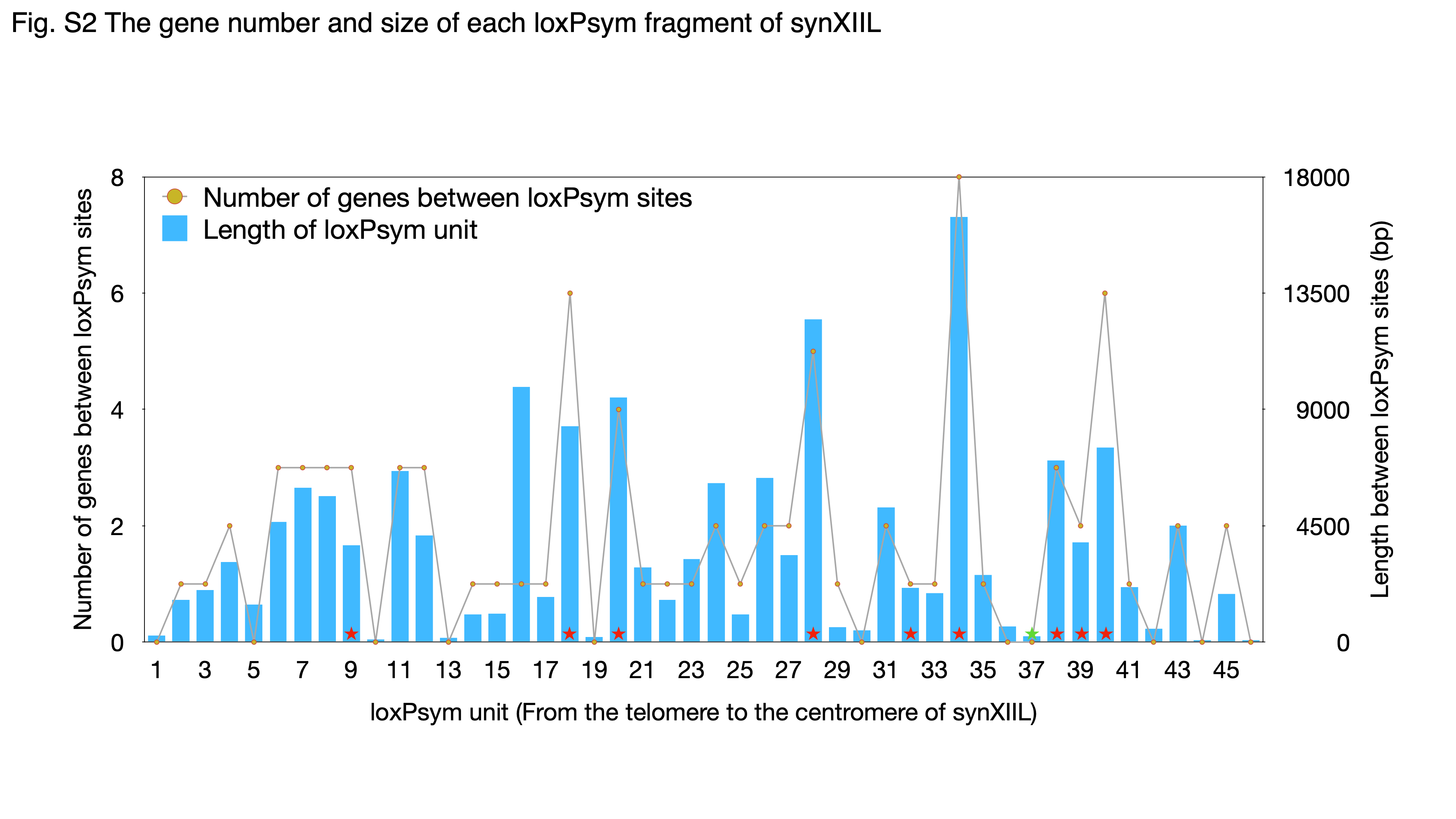
**

The number of genes encoded by each LU is counted and shown as dot plot, along with the length of each LU shown as bar chart. Red stars indicate essential gene(s) containing LUs, and green star indicate centromere-containing LU. LUs are numbered in a telomere to centromere direction.

**Fig S3. Map of the Cre plasmid used in this study.
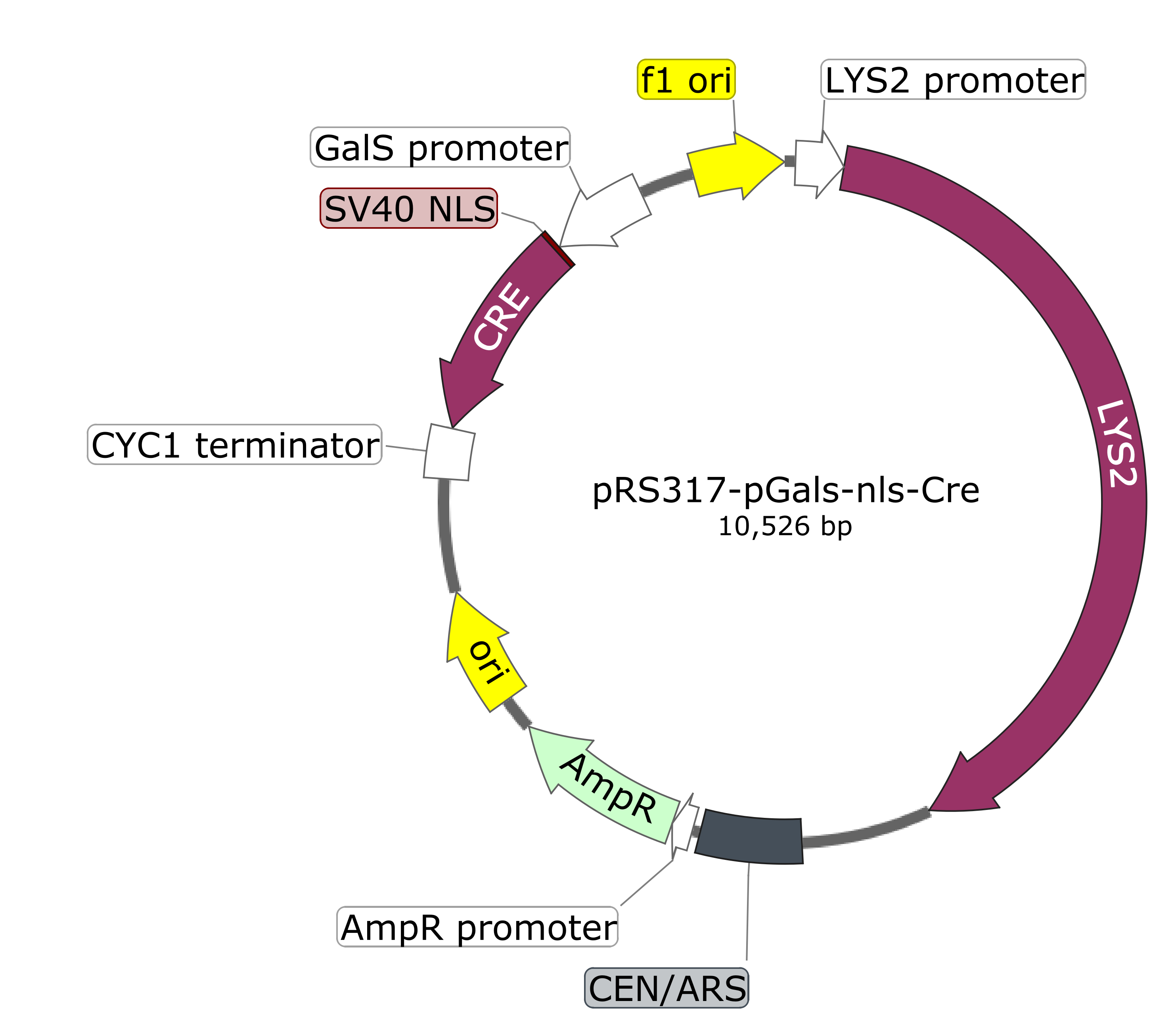
**

Functional elements in this plasmid are shown and plasmid sequence can be found in Table S2.

**Fig S4. An example of PCRtag analysis
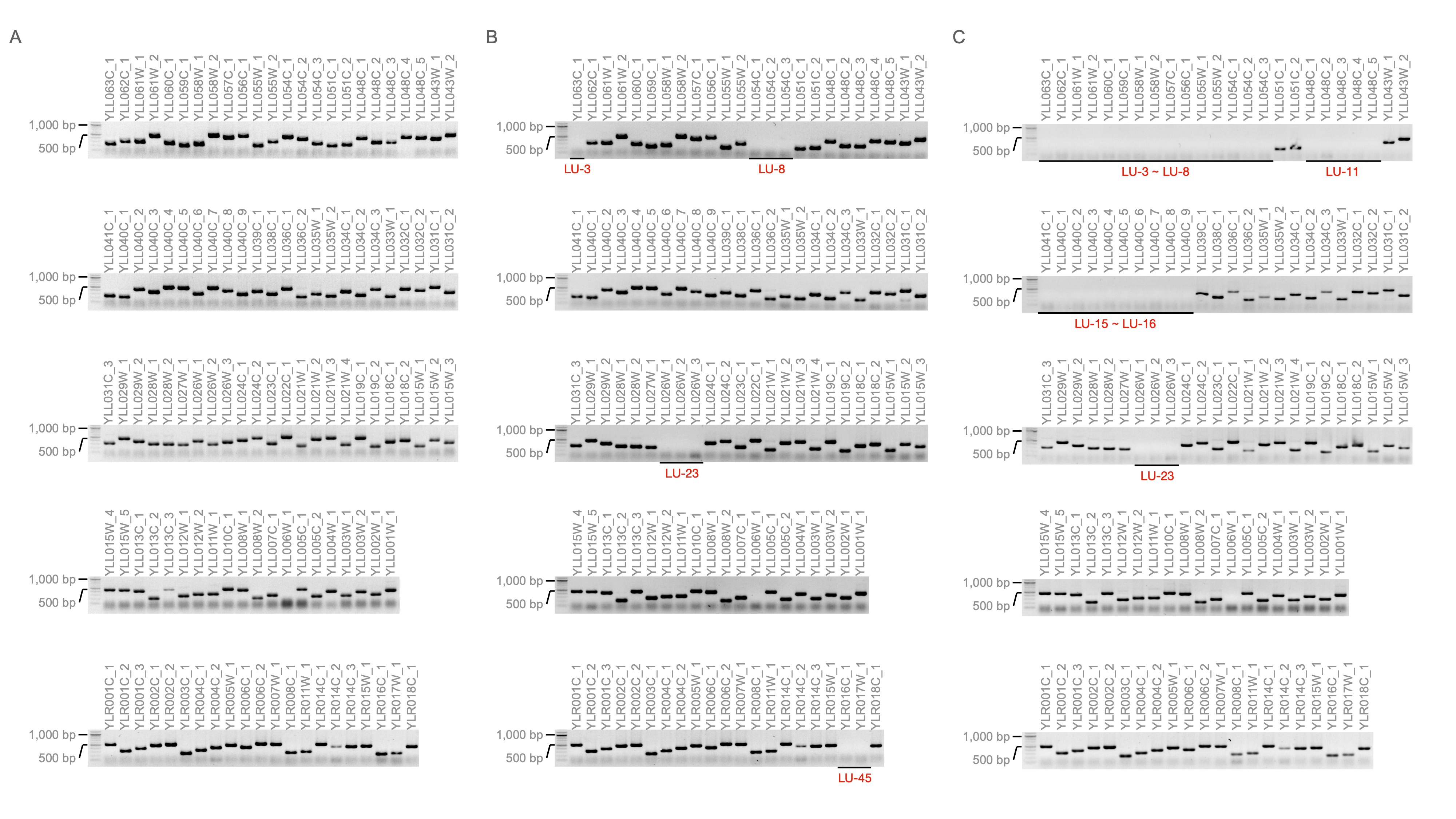
**

PCRtag analysis results of synXIIL (A), ZLY297 (B) and ZLY298 (C) are shown. The name of each amplicon is labeled above the corresponding lane and 2-log DNA marker was used. The deleted LUs in ZLY297 and ZLY298, as indicated by PCRtag analysis, are shown in red and are consistent with the results showed in Fig. 2A. It should be noted that some LU contain no PCRtags, so the deletion of these LUs cannot be indicated by PCRtag analysis.

**Fig S5. Stability of URA3 integrated in synXIIL without the expression of Cre
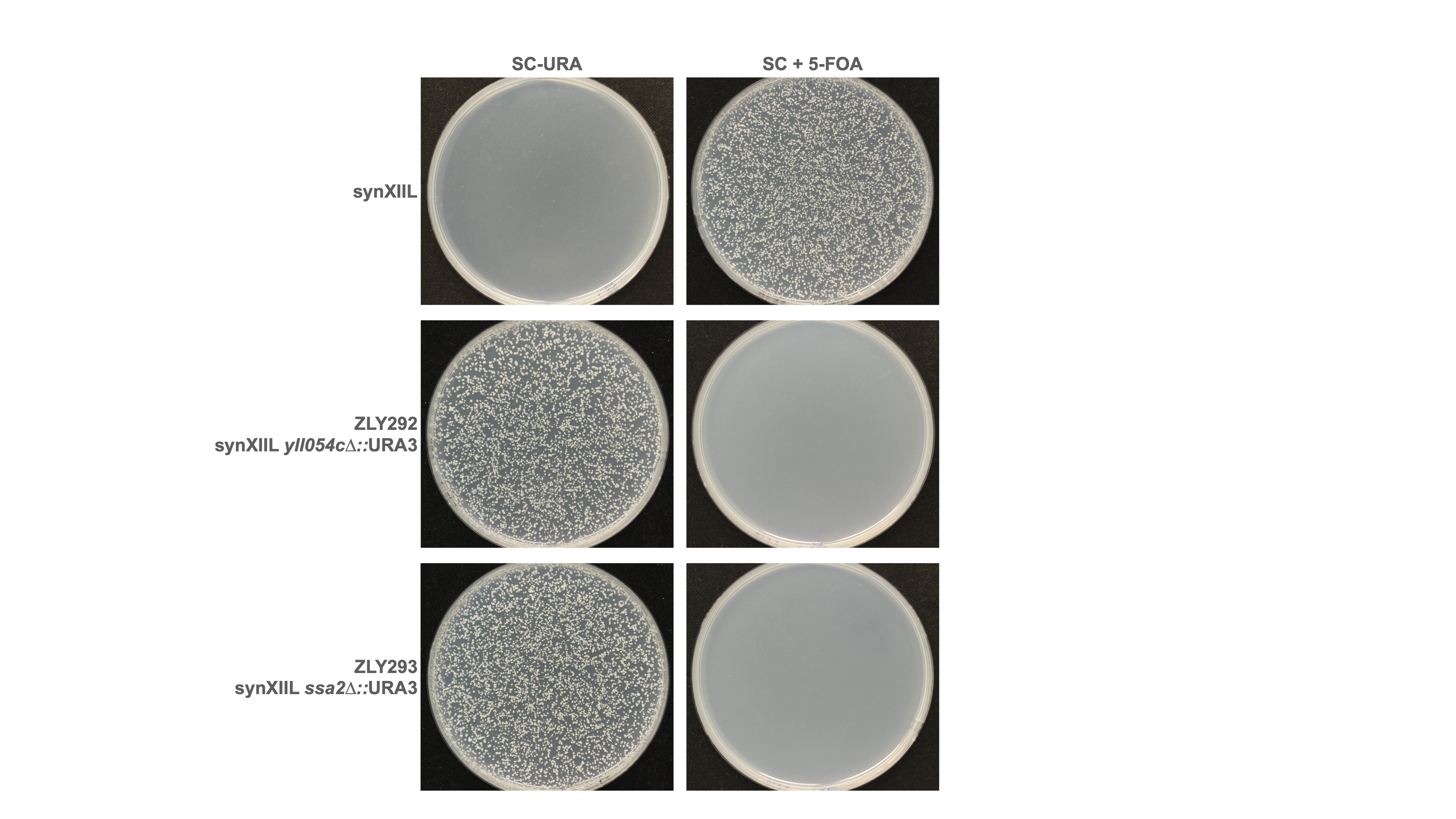
**

2 X 10^4^ cells of indicated strains were plated onto SC-URA plates or SC plates supplemented with 1 g/L 5-FOA (SC + 5-FOA). Images were taken after incubation of plates at 30℃ for 48 hrs. For ZLY292 or ZLY293, no colonies appeared on the SC + 5-FOA plate, indicating that the URA3 gene integrated in synthetic chromosome is stable without the expression of Cre (with a loss rate lower than 10^-4^).

**Fig S6. Genome rearrangements in ZLY294-ZLY298
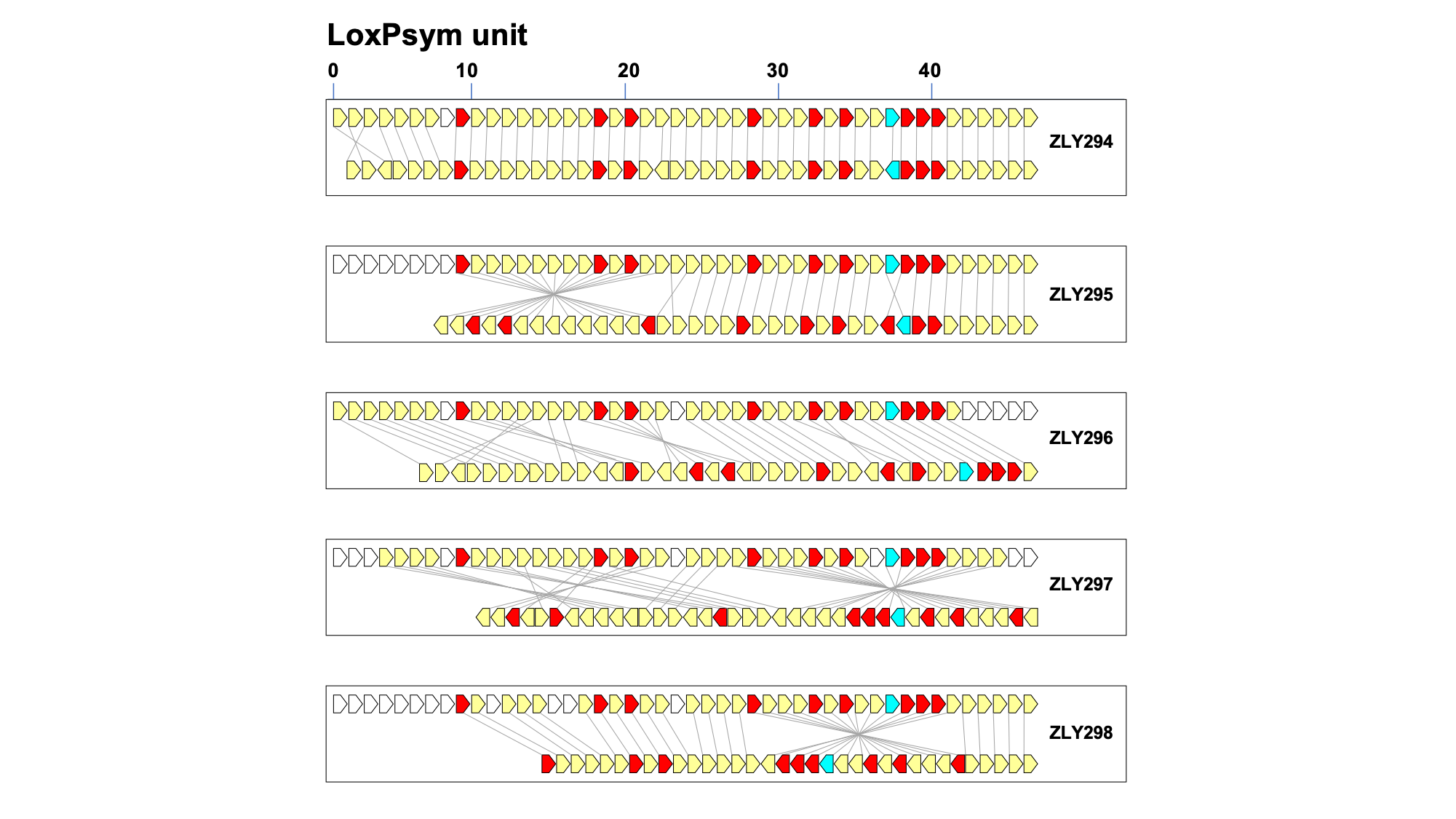
**

Genomes are assembled based on PCRtag analysis and Nanopore sequencing results. Each loxPsym unit is shown as a pentagon with direction. Essential gene(s) containing LUs are shown in red and centromere-containing LU are shown in cyan. Deleted LUs are shown as white pentagons. Corresponding LUs are connected by lines between the parent strain and SGC strain.

**Fig S7. Genome rearrangements in ZLY299-ZLY303
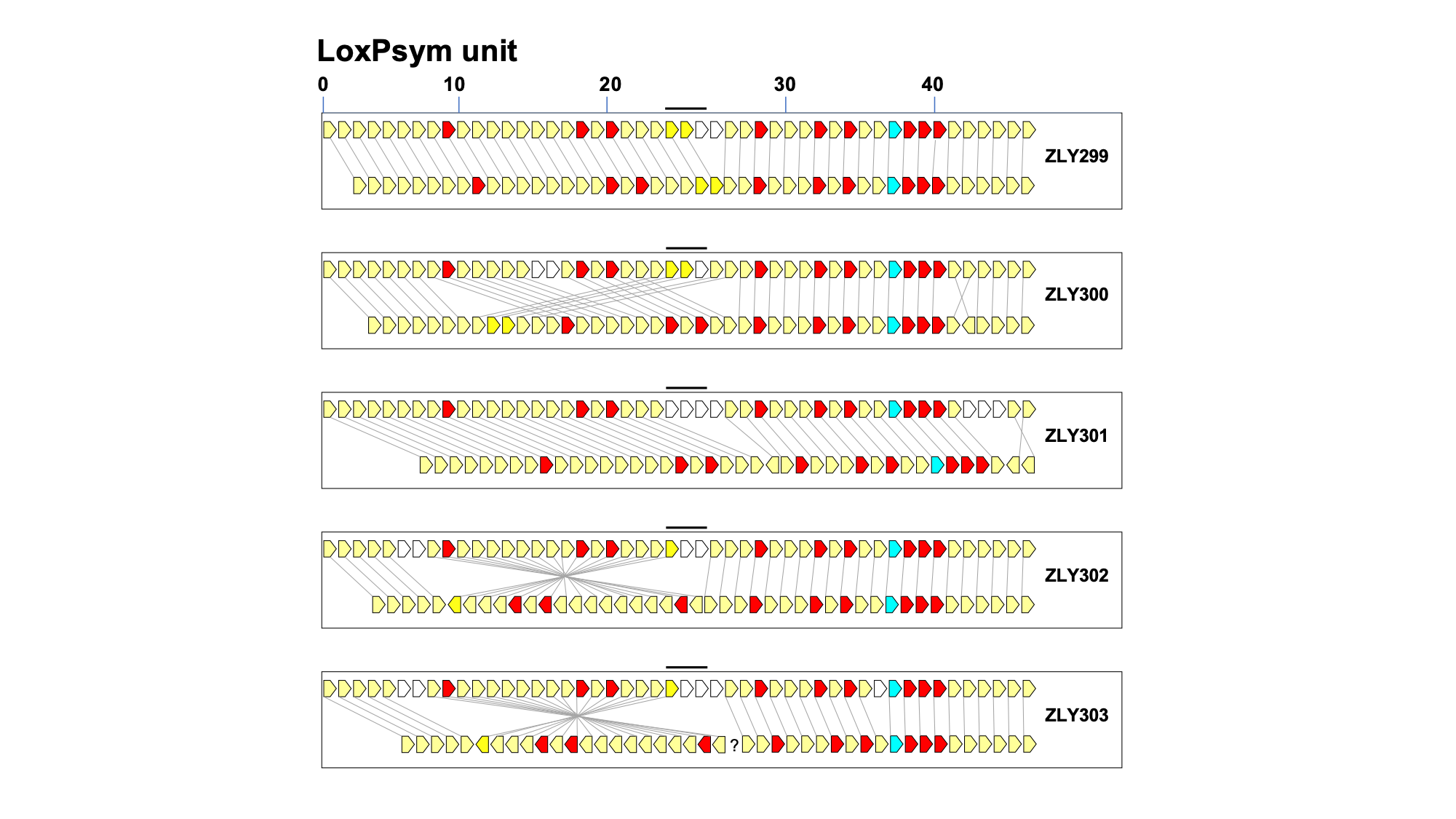
**

Similar to Fig. S6, except for here the short black line indicates the site of loxPsym units 24-a, 24-b and 24-c, which are generated by integrating *ssa2*△::URA3 into LU-24.


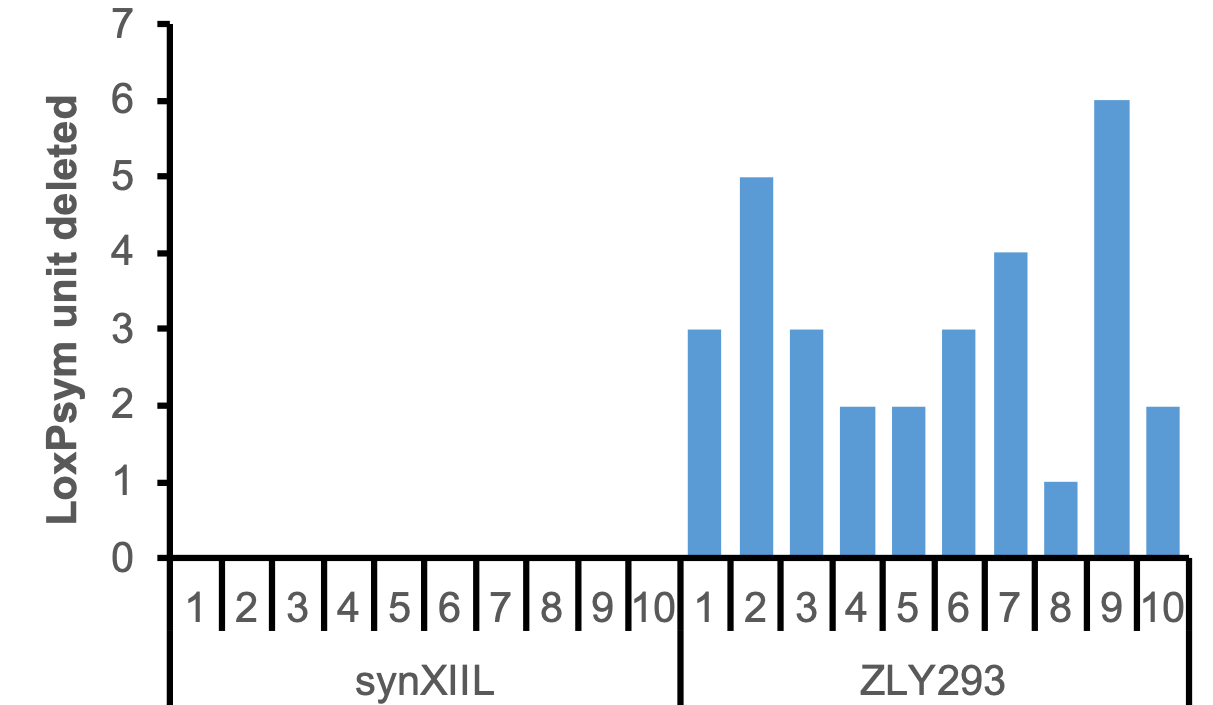
**Fig S8. PCRtag analysis of SCRaMbLEd strains with or without URA3 integration and 5-FOA selection.**

SCRaMbLE was induced for synXIIL and ZLY293 as described in the methods section. After SCRaMbLE, synXIIL was plated onto SC medium and ZLY293 was plated onto SC + 5-FOA plate to selected the URA3 deleted strains. 10 colonies were picked randomly for each strain and subjected to PCRtag analysis. The number of deleted loxPsym units identified by PCRtag analysis for each colony is shown.

**Fig S9. The assembly and verification of eArray**

1. Seven DNA-segments were amplified out from the synXIIL genome and assembled together with a modified pRS413 plasmid backbone in a single step homologous recombination event. URR1 and URR2 are two random sequences that share no similarity with the yeast genome. They are used as a landing pad for amplified sequences. Detailed information of encoded genes can be found in Fig. S5.
2. PCRtag analysis to confirm the presence of designed sequences. This plasmid was assembled in a wild type BY4742 strain, therefore the synthetic PCRtags designed in Sc2.0 can be used to distinguish between plasmid and genomic sequences.
3. Junction PCR analysis to confirm the assembly of segments in the designed order. All eight junctions across inserted segments and plasmid backbone were amplified and amplicon sizes were verified.
4. Restriction enzyme digestion to confirm correct plasmid assembly. The plasmid was transplanted from yeast into *E. coli* and then purified using a plasmid miniprep kit. DNA was run on a 0.8% agarose gel after restriction enzyme digestion to verify the presence of segments of correct size. The expected sizes of segments can be found in Fig. S9F.
5. The distribution of the three selected enzymes restriction sites on the eArray, shown with their exact locations.
6. The simulation of eArray restriction digestion. The exact sizes of digested fragments are listed. The gel fragment running sizes are shown in grey.
7. Dissection results of heterodiploid strains with single essential gene deletion and eArray. Images were taken after 36 hrs of growth on YPD medium at 30℃ after dissection.
8. Serial dilution of haploid strains with single essential gene deletion and eArray. Images were taken after 36 hrs of growth on YPD medium at 30℃ after plating.

**Fig S10. PCRtag analysis to confirm the deletion of LU-20
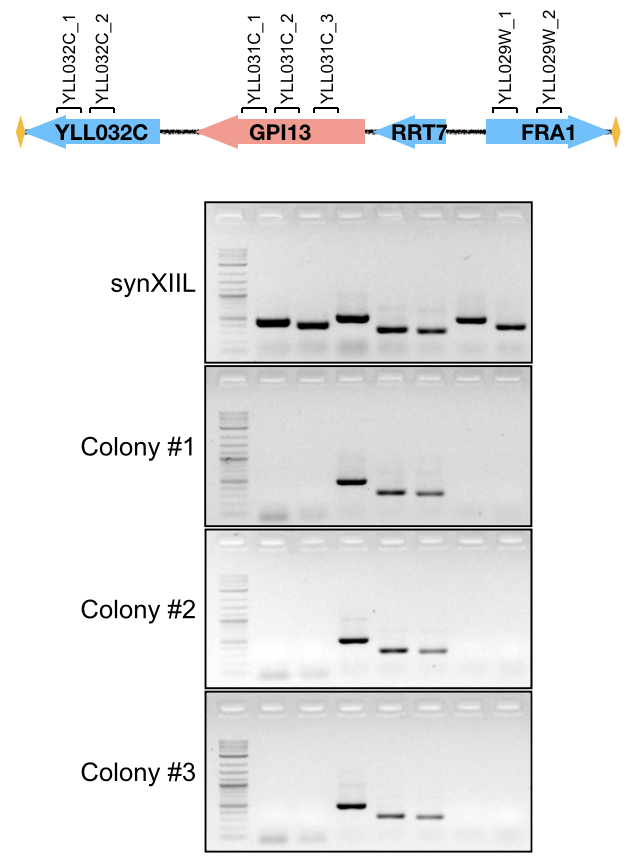
**

There are seven PCRtags in LU-20. The three PCRtags in GPI13 can be amplified out in all strains, demonstrating the presence of synthetic GPI13 on the eArray besides synXIIL. The other four PCRtags located on nonessential genes can be used to indicate the presence of LU-20 in the genome. SynXIIL genome was used as the control template for PCR.

**Fig S11. The deletion boundary reflects the 3D proximity of loxPsym sites
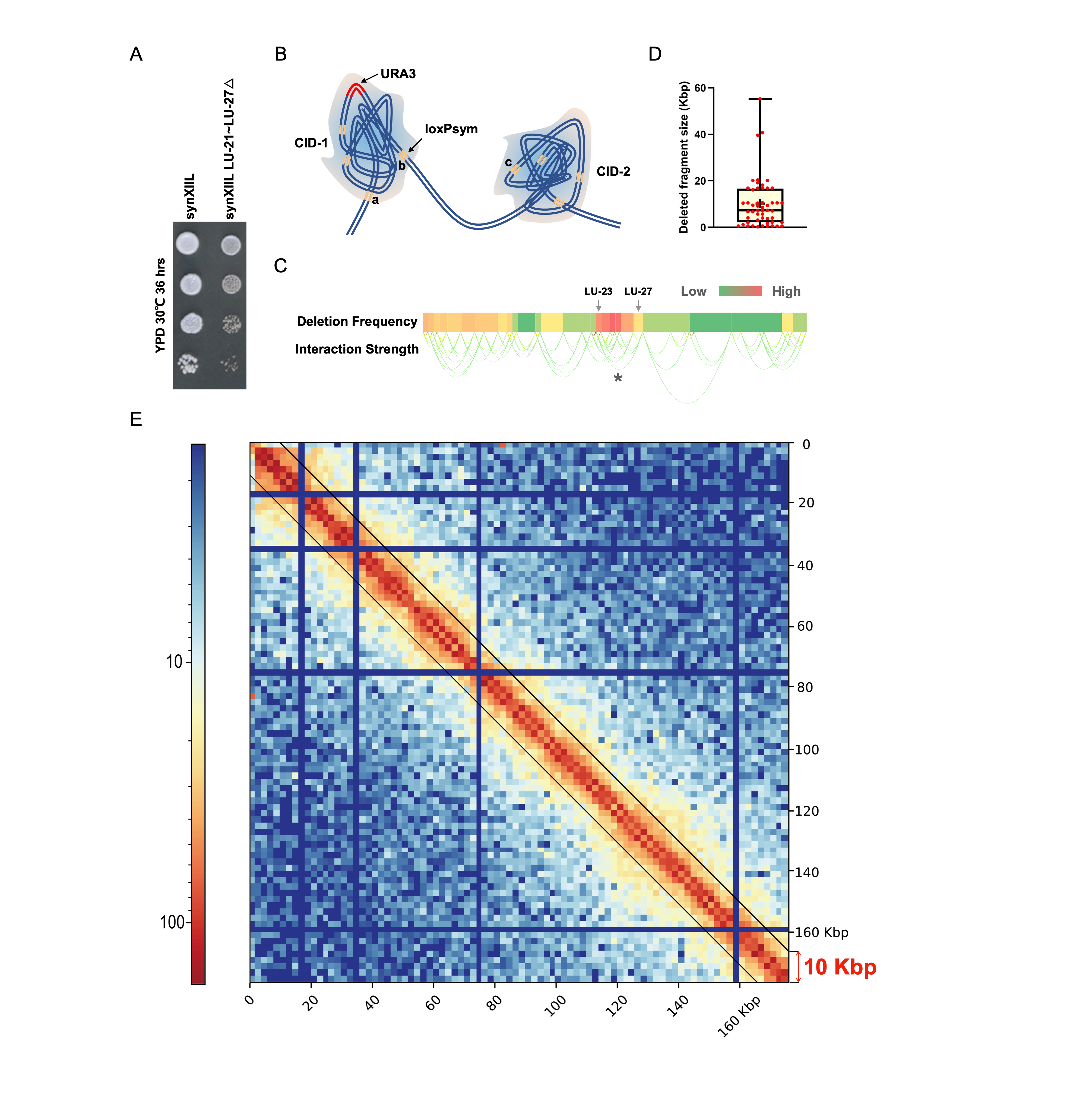
**

1. Growth of the strain without the sequences between GPI13 and DPS1. All genes between GPI13 and DPS1 were inactivated by the deletion from 68,720 bp to 97,384 bp (corresponding to the sequence from LU-21 to LU-27). 10-fold serial dilution was performed for this strain and the parent synXIIL strain.
2. LoxPsym sites located in the same chromosome interaction domain (CID) are more likely to mediate recombination. If we using 5-FOA to select the URA3 deleted strains, most strains we selected will use the loxPsym sites located in CID-1 but not CID-2.
3. The correlation between LU deletion and loxPsym site interaction. The deletion frequency of each LU in Fig. 3C was counted and shown collectively as a heatmap. Green represents low deletion frequency and red represents high deletion frequency. The heatmap direction is displayed from LU-1 to LU-46, the locations of LU-23 and LU-27 are labeled. Interactions between loxPsym sites detected by Hi-C were connected by lines. Green represents low interaction strength and red represents high interaction strength. The asterisk labels an interaction domain detected by Hi-C that directly flanks the sequence from LU-23 to LU-27.
4. The size distribution of segments deleted by a single SCRaMbLE event in strains shown in Fig. 3C. Deleted LUs that are concatenated were regarded to be deleted by a single SCRaMbLE event.
5. Normalized contact map (bin size, 2 kb) of synXIIL. The color scale is shown on the left. Coordinates are labeled on the bottom and right axes. The average interaction range is around 10 kbp as labeled in red.

**Fig S12. Genome rearrangements in iterative SGC strains
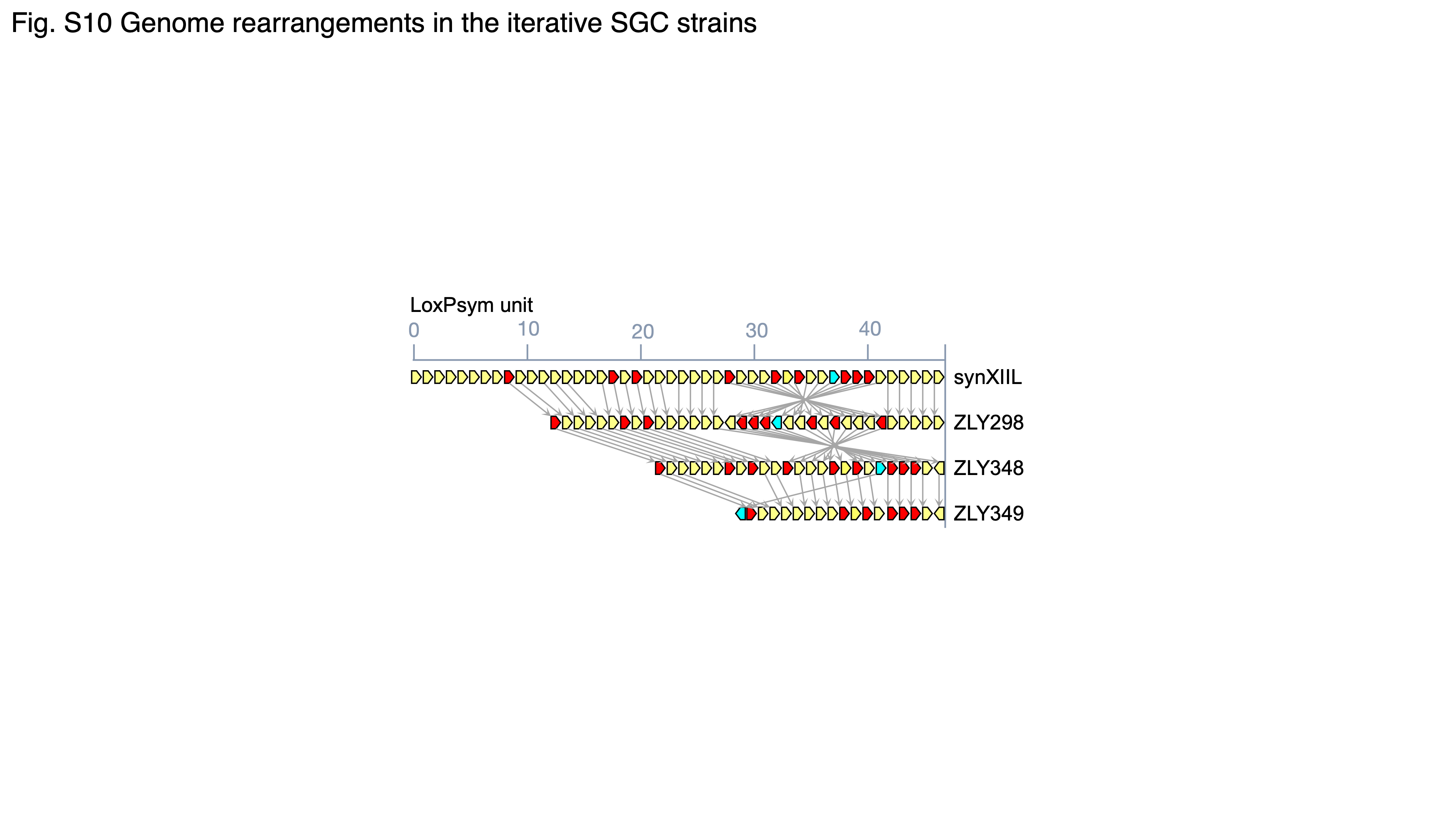
**

Each loxPsym unit is shown as a pentagon with direction. The essential-gene-containing LUs are shown in red and the centromere-containing LU are shown in cyan. Corresponding LUs are connected by short lines with arrowheads between the parent strain and SGC strain.

**Fig S13. The growth of SGC strains on YPD 30**℃**
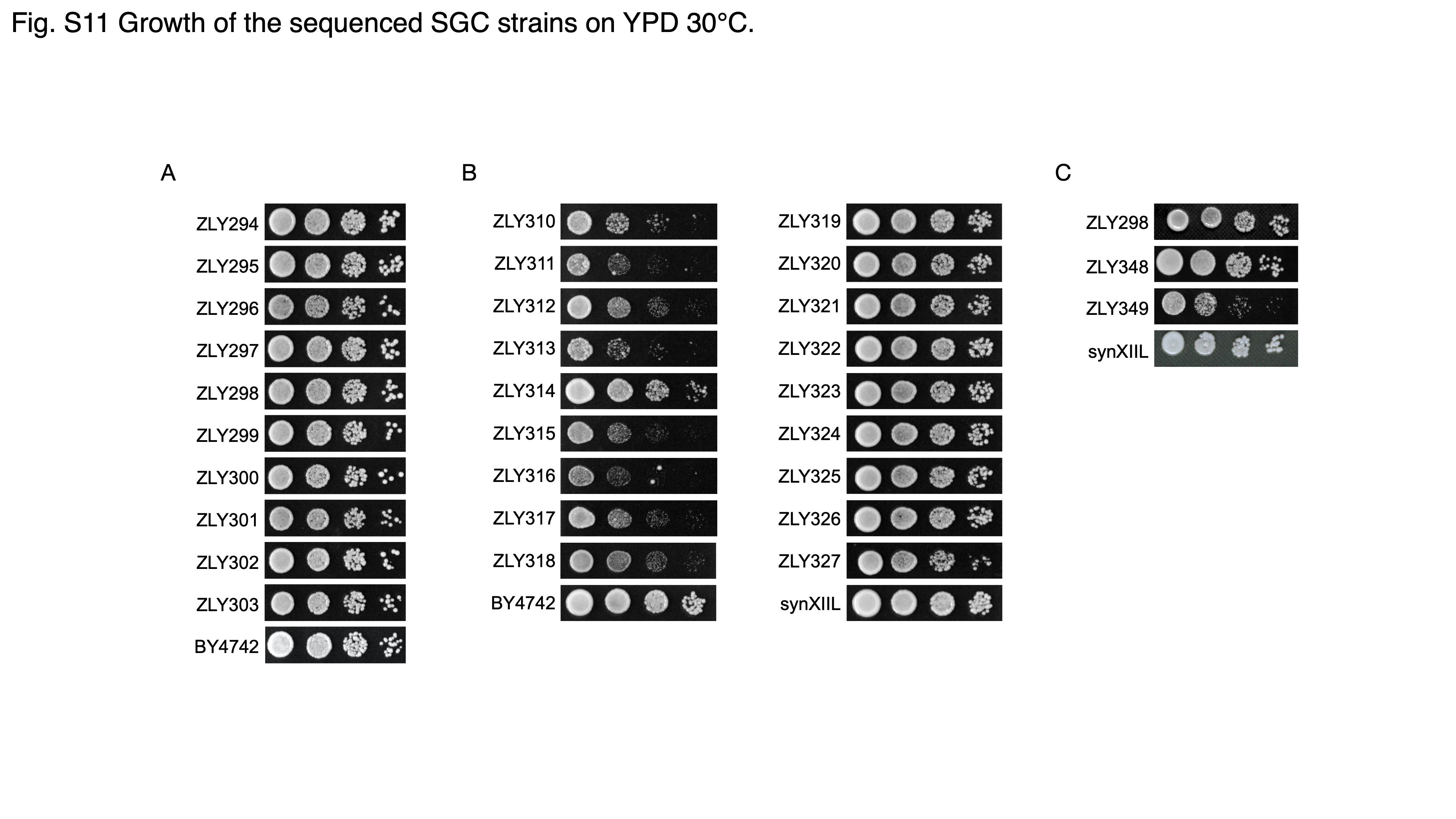
**

10-fold serial dilution on YPD medium was conducted for all SGC strains generated in this study (A shows the strains used in Fig. 2A and 2B, B shows the strains used in Fig. 3C, C shows the strains used in Fig. 4A). The pictures were taken after 36 hrs growth at 30℃.

**Fig S14. Competition among synXIIL, BY4742 and eArray strain with single essential gene deletion.**

The percentage of cells in cocultures of synXIIL, BY4742 and eArray strain with single essential gene deletion in ChrXII was determined at indicated times. 0 in the horizontal axis represents the starting time of cocultures when equal number of cells of the three strains were added into 3 mL YPD medium. The coculture was diluted to OD_600_=0.1 every 24 hrs. 72 on the horizontal axis represents the end time of the third round of 24-hour coculture. Around 300 cells were plated onto YPD plates for each coculture at indicated time. After 48-hour growth at 30℃, the YPD plates were replicated onto SC-HIS to identify the eArray containing colonies and onto SC-LEU to identify the synXIIL colonies. Three independent colonies were tested for each strain. The mean value and standard deviation of percentages are shown.
