# Supplementary material for: Compacting a synthetic yeast chromosome arm
Source: Genome Biol. 2021 Jan 4;22:5. doi: 10.1186/s13059-020-02232-8 (PMC7780613; doi:10.1186/s13059-020-02232-8)
Supplement: Supplementary file 6 — Additional file 6. Review history. [file 13059_2020_2232_MOESM6_ESM.docx]

Review History

**First round of review**

**Reviewer 1**

**Are you able to assess all statistics in the manuscript, including the appropriateness of statistical tests used?**

There are no statistics in the manuscript.

**Comments to author:**

The manuscript by Luo/Yu et al. is an exciting and high-quality publication taking the high-profile topic of synthetic genomics into new territory where a significant portion of a eukaryotic chromosome is minimised via clever synthetic methods. This work represents a significant 'first' as those working on making synthetic eukaryotic genomes (especially those working on the Sc2.0 genome for Baker's yeast) have been long aiming to use the synthetic approach, along with in-built recombination to define minimal eukaryotic genomes. This work now demonstrates the first steps of that, making significant reductions to Chr 12 of Baker's yeast, via deletion of non-essential genes from a synthetic version of that chromosome. It is exciting and unique work that I think is a good fit for the readership of this journal, while also probably being a bit different to what they are used to.

Overall, I found that the quality of the experiments, the design of the work and the presentation of the results was of a very high standard. The paper is close to being publication standard because of this. However, I will recommend minor corrections due to a few small questions and comments I have (see below) and because I think that the written English could do with some improvement in many places. The written english is not terrible, but many phrases and word choices were not quite right and could be improved. I notice that two of the authors are based in England, so I suggest that they take the lead in rewriting the sections where the choice of words/phrase isn't quite right. To help with that I attach my copy of the manuscript PDF with some of the words/sentences that are not quite right highlighted. I note that there are many more than these ones I highlighted, so best if the authors re-read the entire manuscript.

Minor points to revise and comments.

1. English writing - see above

2. The first part of the abstract assumes lots of knowledge most readers won't have - e.g JCVI, synthetic yeast genome project, SCRAMBLE acronym. - this should be altered to keep in mind that the abstract needs to be read by a very broad audience.

3. Description of the design of eArray is lacking. There is a sentence on YeastFab that assumes people know what this is and how it works. I recommend that the authors significantly increase the text used to describe eArray design and potentially include an eArray design and content figure within the main set of Figures.

4. The validation of the eArray design by spore tetrads was very nice but I still wondered how well the eArray-based gene can perform its task compared to the synXIIL based gene. Is it possible to use the deletion rates to assess this? I would expect that a working eArray would lead to the red LUs in Fig 3 being deleted with the same frequency as the grey LUs, but from the figure it looks like there is still less loss of red LUs. Perhaps the eArray-based essential genes are 'good enough' to complement a KO to allow survival, but are not 'good enough' (correctly expressed) to replace the chromosomal-based ones in a direct competition.

5. The section on page 7 using Hi-C data and talking about the 3D genome was not convincingly described and felt out of place in this manuscript. The authors should consider improving the description or perhaps reassessing what benefit it has to add this.

6. Figure 5C has boxes highlighted in green and red but these colours are not described in the figure legend.

**Reviewer 2**

**Are you able to assess all statistics in the manuscript, including the appropriateness of statistical tests used?**

Yes, and I have assessed the statistics in my report.

**Comments to author:**

In this manuscript, the authors developed a method based on SCRaMbLE to compact the synthetic chromosome arm of chromosome XII constructed by the Sc2.0 consortium. By creating an array containing essential genes (eArray), the authors demonstrated that large segments of the synXIIL can be deleted while retaining reasonable level of cellular fitness in the strains generated. Furthermore, the authors analysed the deletion events to find the correlation between the 3D proximity of loxPsym sites and the likelihood of recombination between these sites. This work is a great demonstration of the ability of using SCRambLE in Sc2.0 to compact the genome of Saccharomyces cerevisiae, which would have been difficult to achieve by rational design using classical genomic techniques. Overall, the content of the manuscript is well-organized and insightful. However, there are limitations to the technique developed and the manuscript could be further proofread. The authors should address the following points when revising the manuscript.

Major points:

1. One major limitation of the method developed is the need for insertion of URA3 markers. To facilitate compaction of synXIIL, the authors inserted URA3 markers at LU-8, LU-24 and LU-20 followed by 5-FOA selection to enrich the population. As acknowledged by the authors in the Discussion section, careful experimental design is needed when choosing the location of the URA3 marker. While this selection guarantees the isolation of strains with at least one deletion, it restricts the versatility and generalizability of the method, as the deletions would be clustered around the URA3 marker and cause a bias. How critical is the insertion of URA3 marker in the success of chromosome compaction? How rare are deletions during SCRaMbLE where URA3 markers are required for a pre-selection? The authors could include some background on the frequency of deletion events during SCRaMbLE to support the need for inserting URA3 markers.

2. The possible choices of marker insertion sites will increase exponentially with the number of synthetic chromosomes. Hence, it may be difficult in future with Sc2.0 strains with multiple synthetic chromosomes (page 7, line 56) to iteratively insert URA3 to progressively compact the genome. Are there ways to overcome the need for URA3 insertion to facilitate the genome compaction process? Without markers, the locations of the deletion events would also be less restricted and less biased. One possibility is to combine SCRaMbLE with lab adaptive evolution to increase the chances of deletion events over many generations, such that clones isolated from the final population (e.g. on streaked plates) will likely have at least one deletion. The authors should discuss the feasibility of incorporating lab adaptive evolution or propose other possible markerless methods, as this would be important for genome compaction of multi-synthetic chromosome strains or even the complete Sc2.0 strain.

3. Page 4, line 48: The authors should briefly explain PCRtag analysis to readers who are unfamiliar with the Sc2.0 project.

4. Page 4, line 59-60: ZLY294 and ZLY299 have only a deleted segment flanking the URA3 marker, which could be due to spontaneous intra-chromosomal recombination. The authors should acknowledge this possibility and also provide data of a control without Cre expressed to estimate the frequency of spontaneous URA3 deletion.

5. Page 8, line 41: Since the minimal genome is situational, the authors could apply the technique to identify the compacted genome under another growth condition. If there are genes in synXIIL that confer tolerance (e.g. high salt, high temperature), a compacted genome that differs from that isolated under growth in YPD may be identified, and hence demonstrate the importance of the genes in responding to the stress applied. This would lend support to the authors claim that "every gene should have the possibility to be SCRaMbLEd individually to allow the dissection of the essentiality of individual gene".

6. Page 12, line 37-47: Cre expression was induced only with galactose and no estradiol was required, which is significantly different from most reported SCRaMbLE experiments. The authors should describe the SCRaMbLE system and plasmid used and provide the plasmid map as supplementary information.

Minor points

1. The manuscript should be further proofread to rectify grammatical and typographical errors. Below are some suggestions:

2. Page 3, line 19: Change "Compare" to "Compared"

3. Page 3, line 60: Change "And what's more" to "Furthermore".

4. Page 4, line 1: Change "events be prohibited" to "events prohibited".

5. Page 4, line 11: Throughout the manuscript, consider changing "fragments" to "segments" when referring to deleted LUs.

6. Page 4, line 16: Delete "happened"

7. Page 4, line 16: Change "quickly" to "quick"

8. Page 4, line 29 and Page 13, line 39: Change "Totally" to "In total"

9. Page 4, line 44: It might be redundant to specify "see methods for the details of SCRaMbLE induction". Consider deleting it.

10. Page 5, line 11: Change "an compensatory" to "a compensatory"

11. Page 5, line 14: Throughout the manuscript, change all "essential gene containing LU" to "essential-gene-containing LU"

12. Page 5, line 33: Change "sequences that 500 bp" to "sequences that are 500 bp"

13. Page 6, line 25: Please clarify if the figure is Fig. 1E or Fig 2E.

14. Page 6, lines 47-48: Change the three "totally" to "total"

15. Page 6, line 60 and Page 7, line 2: Change "at the presence" to "in the presence"

16. Page 7, line 11: Change "flanked LU24 is" to "flanking LU24 are"

17. Page 7, line 11 and Page 10, line 23: Change "can not" to "cannot"

18. Page 9, line 2: Change "out of the totally" to "out of the total"

19. Page 10, line 11: Change "URA3 integrated" to "URA3-integrated"

20. Page 10, line 17: Change "chose" to "choice"

21. Page 10, line 55: Change "sequences been deleted" to "sequences deleted"

22. Page 10, line 55: Change "we shown" to "we have shown"

23. Page 12, line 40: Please revise "sub" to a more suitable term

24. Page 14, line 30: Please replace "XXX" with the accession number

25. Page 16, line 21: Change "centromere containing" to "centromere-containing" throughout the manuscript (Fig. S2, S3 and S10 too)

26. Figures 2A, 2B, 3B, 4A: Please change cyan to another colour for the centromere-containing LU. Cyan does not stand out well next to gray.

**Authors Response**

**Point-by-point responses to the reviewers’ comments:**

Response to Reviewer #1:

The manuscript by Luo/Yu et al. is an exciting and high-quality publication taking the high-profile topic of synthetic genomics into new territory where a significant portion of a eukaryotic chromosome is minimised via clever synthetic methods. This work represents a significant 'first' as those working on making synthetic eukaryotic genomes (especially those working on the Sc2.0 genome for Baker's yeast) have been long aiming to use the synthetic approach, along with in-built recombination to define minimal eukaryotic genomes. This work now demonstrates the first steps of that, making significant reductions to Chr 12 of Baker's yeast, via deletion of non-essential genes from a synthetic version of that chromosome. It is exciting and unique work that I think is a good fit for the readership of this journal, while also probably being a bit different to what they are used to.

Overall, I found that the quality of the experiments, the design of the work and the presentation of the results was of a very high standard. The paper is close to being publication standard because of this. However, I will recommend minor corrections due to a few small questions and comments I have (see below) and because I think that the written English could do with some improvement in many places. The written english is not terrible, but many phrases and word choices were not quite right and could be improved. I notice that two of the authors are based in England, so I suggest that they take the lead in rewriting the sections where the choice of words/phrase isn't quite right. To help with that I attach my copy of the manuscript PDF with some of the words/sentences that are not quite right highlighted. I note that there are many more than these ones I highlighted, so best if the authors re-read the entire manuscript.

Minor points to revise and comments.

1. English writing - see above

*The entire manuscript has been revised by a native speaker to improve English.*

2. The first part of the abstract assumes lots of knowledge most readers won't have - e.g JCVI, synthetic yeast genome project, SCRAMBLE acronym. - this should be altered to keep in mind that the abstract needs to be read by a very broad audience.

*We have made changes accordingly and made it meet the requirement that the Abstract section should not exceed 250 words.*

3. Description of the design of eArray is lacking. There is a sentence on YeastFab that assumes people know what this is and how it works. I recommend that the authors significantly increase the text used to describe eArray design and potentially include an eArray design and content figure within the main set of Figures.

*A detailed description on the design of eArray is added in the text. The design and content of the eArray has been included as Fig. 3A. The sequence of eArray is provided in Table S2.*

4. The validation of the eArray design by spore tetrads was very nice but I still wondered how well the eArray-based gene can perform its task compared to the synXIIL based gene. Is it possible to use the deletion rates to assess this? I would expect that a working eArray would lead to the red LUs in Fig 3 being deleted with the same frequency as the grey LUs, but from the figure it looks like there is still less loss of red LUs. Perhaps the eArray-based essential genes are 'good enough' to complement a KO to allow survival, but are not 'good enough' (correctly expressed) to replace the chromosomal-based ones in a direct competition.

*We thank the reviewer for this suggestion. Because the essential gene containing LUs often includes other nonessential genes, which may result in severe growth defects when deleted (such as MMM1 and RTT109 referred in manuscript), and the gene content of each LU is different, using the deletion rates of LUs to assess the function of eArray may not reflect the goodness of eArray-based essential genes.*

*To address the reviewer's concern, we performed a coculture experiment as described in supplementary material (Fig. S14). In this experiment, the essential gene deleted haploid strains carrying eArray obtained by tetrads dissection was co-cultured with synXIIL and BY4742 to test whether the competition ability of these strains are comparable to synXIIL or BY4742. The competition results after 72 hrs coculture suggested the eArray-based essential genes are not 'good enough' to replace the chromosomal-based ones in a direct competition. The presence of additional copy of other essential genes except the deleted ones, the stability of eArray and the loss of native regulation may contribute to the lower fitness in competition.*

*Since the current design of eArray meets our requirement for genome compaction, although not perfect, as described above, in this manuscript, we didn't further address this question. We have included this data in Discussion section.*

5. The section on page 7 using Hi-C data and talking about the 3D genome was not convincingly described and felt out of place in this manuscript. The authors should consider improving the description or perhaps reassessing what benefit it has to add this.

*We have made changes accordingly and improved the description of Hi-C data. The reason why we include this data is to explain the phenomenon that the deleted LUs flanked LU24 are mostly restricted from LU-23 to LU-27 (Fig. 3C). A discussion about future applications of the correlation is also included in Discussion.*

*The updated description of this data is as following:*

*Although the presence of the eArray expands general compacting ability, the deleted LUs flanked LU-24 are mostly restricted from LU-23 to LU-27 (Fig. 3C). This cannot be a result of the synthetic lethal effect, as the sequence from LU-18 to LU-28 is deleted in strain ZLY315 by SGC (Fig. 3C) and the sequences from LU-21 to LU-27 could be deleted using the CRISPR/Cas9 method (Fig. S11A).*

*Cre-loxP mediated deletion requires the formation of a synapsed structure by four Cre recombinases, pairs of which recognize a loxPsym site [41]. If two loxPsym sites (for example, the a and b loxPsym sites in Fig. S11B) are located in the same chromosomal interacting domain (CID), the Cre recombinases binding them are more likely to come into contact with each other to mediate tetramer formation due to their 3D proximity, compared to the two loxPsym sites located in different CIDs (for example, the a and c loxPsym sites in Fig. S11B). Therefore, the closer the two loxPsym sites are within the 3D genomic structure, the more likely they are to mediate deletion events. Similarly, the restricted deletion range we observed here may reflect the 3D proximity of loxPsym sites from LU-23 to LU-27. To test this, we extracted and analyzed the 1-170 kbp Hi-C data of synXII from our previous publication [42]. The sequence from LU-23 to LU-27 perfectly matches a CID detected by our Hi-C analysis (labeled by an asterisk in Fig. S11C). This is to say, the loxPsym sites outside LU-23 to LU-27 are in different CIDs, thus they are hard to mediate URA3 deletion.*

*A previous work in mice also showed results similar to us, suggesting that Cre recombination efficiency is inversely proportional to the genetic distance between loxP sites [43]. Given this, we next asked whether this is applicable to other regions in synXIIL. Since the strains in Fig. 3C were exposed to Cre activity for only two hours, we considered deleted, concatenated LU the result of single deletion event. Thus, according to our hypothesis, most deletion events we detected in these strains should have sequence lengths equal to or shorter than the lengths of CIDs. The size distribution of deleted segments in these strains were calculated and shown in Fig. S11D, the relative short deletion length (mean=10 kbp, medium=7 kbp) is comparable to the limited intra-chromosomal interaction range of synXIIL (about 10 kbp at each side, Fig. S11E), which is also consistent with the Micro-C results reported by Hsieh and colleagues [44], that CIDs in yeast are typically 2-10 kbp in length. These results further support our hypothesis that loxPsym sites located in the same CID more favourably mediate recombination due to their 3D proximity.*

*In summary, a positive correlation between the proximity of loxPsym sites in 3D structure and the likelihood of these loxPsym sites meditating a deletion event is revealed by our data, suggesting the 3D contact information of loxPsym sites can potentially be used to guide the selection of URA3 integration sites. It will be of interest to test this observation in more complex situations, for example, in a multi-synthetic chromosome background, to see whether it will be easier to delete more LUs in a single SGC process if URA3 is integrated into a CID covering longer sequence.*

6. Figure 5C has boxes highlighted in green and red but these colours are not described in the figure legend.

*These boxes have been removed*

Response to Reviewer #2:

In this manuscript, the authors developed a method based on SCRaMbLE to compact the synthetic chromosome arm of chromosome XII constructed by the Sc2.0 consortium. By creating an array containing essential genes (eArray), the authors demonstrated that large segments of the synXIIL can be deleted while retaining reasonable level of cellular fitness in the strains generated. Furthermore, the authors analysed the deletion events to find the correlation between the 3D proximity of loxPsym sites and the likelihood of recombination between these sites. This work is a great demonstration of the ability of using SCRambLE in Sc2.0 to compact the genome of Saccharomyces cerevisiae, which would have been difficult to achieve by rational design using classical genomic techniques. Overall, the content of the manuscript is well-organized and insightful. However, there are limitations to the technique developed and the manuscript could be further proofread. The authors should address the following points when revising the manuscript.

Major points:

1. One major limitation of the method developed is the need for insertion of URA3 markers. To facilitate compaction of synXIIL, the authors inserted URA3 markers at LU-8, LU-24 and LU-20 followed by 5-FOA selection to enrich the population. As acknowledged by the authors in the Discussion section, careful experimental design is needed when choosing the location of the URA3 marker. While this selection guarantees the isolation of strains with at least one deletion, it restricts the versatility and generalizability of the method, as the deletions would be clustered around the URA3 marker and cause a bias. How critical is the insertion of URA3 marker in the success of chromosome compaction? How rare are deletions during SCRaMbLE where URA3 markers are required for a pre-selection? The authors could include some background on the frequency of deletion events during SCRaMbLE to support the need for inserting URA3 markers.

*We thank the reviewer to point this issue out. As we referred in the text, previous reports have shown that the rearrangements generated by SCRaMbLE are random and not all SCRaMbLEd strains contain deletion events (PMID: 30765692, 29789541, 29789590, 30224715 and 32054834). Thus, a method facilitating the rapid identification of the SCRaMbLEd strains with deletion events will be helpful for using SCRaMbLE as an efficient genome compaction tool.*

*In addition, we directly compared the frequency of deletion events can be identified with or without the insertion of URA3 marker and 5-FOA selection. We found without selection, none of the 10 randomly isolated yeast clones showed any missing PCRtag. However, 1 to 6 deleted LUs could be identified in the 10 randomly isolated clones with selection. Therefore, it seems necessary to use this URA3-based selection. The results were presented in Fig. S8.*

2. The possible choices of marker insertion sites will increase exponentially with the number of synthetic chromosomes. Hence, it may be difficult in future with Sc2.0 strains with multiple synthetic chromosomes (page 7, line 56) to iteratively insert URA3 to progressively compact the genome. Are there ways to overcome the need for URA3 insertion to facilitate the genome compaction process? Without markers, the locations of the deletion events would also be less restricted and less biased. One possibility is to combine SCRaMbLE with lab adaptive evolution to increase the chances of deletion events over many generations, such that clones isolated from the final population (e.g. on streaked plates) will likely have at least one deletion. The authors should discuss the feasibility of incorporating lab adaptive evolution or propose other possible markerless methods, as this would be important for genome compaction of multi-synthetic chromosome strains or even the complete Sc2.0 strain.

*We think the exponentially increase of possible URA3 integration sites is good for iterative SGC, instead of making it more difficult. Any one of the possible URA3 integration sites can be used in the first round SGC, and some of the sites that are retained after this round of SGC can be used in the next round, until no sites can be used. Several integration sites can be tested in one SGC process. The phenomenon that no colonies can be obtained or all colonies are smaller than its parent after SGC indicates the genes in the same LUs with URA3 integration are required for minimal genomes.*

*Since the gene essentiality is context dependent, it is worth further testing whether the choice of different sites for URA3 integration would result in different minimal genomes, which could provide direct evidences for the context dependence of minimal genome (PMID: 29033457).*

*We have made changes in Discussion as following:*

*First, not all genomes contain deletions after SCRaMbLE. URA3 integration and 5-FOA selection is used in the SGC method to quickly identify strains containing at least one deletion event and enrich the population for reduced genome sizes. Since the URA3-integrated LU will be excluded in the selected genomes, careful experimental design should be applied to its location. In this study, we chose LUs that do not affect robust growth once deleted. Our data also suggested the LUs located in CIDs covering longer sequences will also be good choices. The possible choice of URA3 insertion site will increase exponentially with the number of synthetic chromosomes. Any one of the possible URA3 integration sites can be used in the first round SGC and the sites retained after this round of SGC can be used in the next round, until no more sites can be used. Several integration sites can be tested in one SGC process. The phenomenon that no colonies can be obtained or all colonies are smaller than their parent after SGC indicates genes in the URA3-integrated LU are required in minimal genomes. Since gene essentiality is context dependent, it is worth further testing whether the choice of different sites for URA3 integration would result in different minimal genomes [16].*

*Second, the drawback of SGC is that a URA3-integrated LU will be excluded in the compacted genome. Because the number of rearranged genomes is extremely large after SCRaMbLE, to identify all compacted genomes is a nearly impossible goal using currently known methods. Different compacted genomes may produce a similar biological fitness under defined growth conditions and our method can generate a part of them, as we have shown in Fig. 5 and Fig. S13. Therefore, the bias of SGC is acceptable if our goal is not to obtain all possible minimal genomes. Without URA3 integration and 5-FOA selection, PCRtag analysis can be used to identify strains with deletions if enough colonies are screened. By using this marker-free method, the locations of the deletion events would be less restricted and less biased. However, given that thousands of PCRtags will present in the final Sc2.0 strain, it will be highly labor-intensive to identify clones with reduced genomes after SCRaMbLE. One possibility is to combine SCRaMbLE with lab adaptive evolution to increase the chances of deletion events over many generations, such that clones isolated from the final population will likely have at least one deletion. However, it may take much longer time to obtain suitable clones for use in further rounds of SGC. Other methods that can accurately, sensitively and quickly measure the DNA content in a single cell are needed to screen SCRaMbLEd cells with deletions. "*

3. Page 4, line 48: The authors should briefly explain PCRtag analysis to readers who are unfamiliar with the Sc2.0 project.

*It has been added and example results of PCRtag analysis are included as Fig. S4.*

4. Page 4, line 59-60: ZLY294 and ZLY299 have only a deleted segment flanking the URA3 marker, which could be due to spontaneous intra-chromosomal recombination. The authors should acknowledge this possibility and also provide data of a control without Cre expressed to estimate the frequency of spontaneous URA3 deletion.

*We thank the reviewer to point out this possibility. We have acknowledged this possibility in the manuscript as" Since previous results have shown the high stability of synthetic chromosome [17] and no colonies were identified by directly plating 2 x 104 cells of ZLY292 or ZLY293 without Cre recombinase onto 5-FOA plates (Fig. S5), we considered the deletions in ZLY294 and ZLY299 as the results of Cre mediated deletion but not the results of spontaneous intra-chromosomal recombination, although this possibility cannot be excluded".*

*In addition, as shown in synIII paper (PMID: 24674868), the synthetic chromosome is very stable without Cre expression, no deletions were observed after over 200,000 segment-generations analyzed;*

*We have performed the control experiment by directly plating the URA3 integrated strain without Cre expressed onto 5-FOA plate. We found that no colonies could appear. These data are added in Fig. S5.*

5. Page 8, line 41: Since the minimal genome is situational, the authors could apply the technique to identify the compacted genome under another growth condition. If there are genes in synXIIL that confer tolerance (e.g. high salt, high temperature), a compacted genome that differs from that isolated under growth in YPD may be identified, and hence demonstrate the importance of the genes in responding to the stress applied. This would lend support to the authors claim that "every gene should have the possibility to be SCRaMbLEd individually to allow the dissection of the essentiality of individual gene”.

*This is a very good suggestion and exactly what we want to do next. The simplicity and effectiveness of the SGC process allow the selection and comparison of compacted genomes supporting different growth rates under different conditions, which can be used to clarify the core genomes for viability and the laws of genomic robustness.*

*In this manuscript, we focused on compacting synXIIL in YPD medium. As we shown in Fig.5, genes required for robust growth under high temperature (PML1) or alternative carbon source (SDH2) conditions were deleted in ZLY348, a strain with compacted synXIIL but its growth in YPD medium were largely unaffected. These results provided an indirect evidence that if we applied our procedure under these stress conditions, these two genes should be included in the compacted genome.*

6. Page 12, line 37-47: Cre expression was induced only with galactose and no estradiol was required, which is significantly different from most reported SCRaMbLE experiments. The authors should describe the SCRaMbLE system and plasmid used and provide the plasmid map as supplementary information.

*The SCRaMbLE system has been described in text and plasmid map/sequence are provided as supplementary information Fig. S3 and Table S2.*

Minor points

1. The manuscript should be further proofread to rectify grammatical and typographical errors. Below are some suggestions:

*The entire manuscript has been revised by a native speaker.*

2. Page 3, line 19: Change "Compare" to “Compared"

*Changed*

3. Page 3, line 60: Change "And what's more" to “Furthermore".

*Changed*

4. Page 4, line 1: Change "events be prohibited" to "events prohibited”.

*Changed*

5. Page 4, line 11: Throughout the manuscript, consider changing "fragments" to "segments" when referring to deleted LUs.

*Changed*

6. Page 4, line 16: Delete “happened"

*Changed*

7. Page 4, line 16: Change "quickly" to “quick"

*Changed*

8. Page 4, line 29 and Page 13, line 39: Change "Totally" to "In total”

*Changed*

9. Page 4, line 44: It might be redundant to specify "see methods for the details of SCRaMbLE induction". Consider deleting it.

*Deleted*

10. Page 5, line 11: Change "an compensatory" to "a compensatory”

*Changed*

11. Page 5, line 14: Throughout the manuscript, change all "essential gene containing LU" to "essential-gene-containing LU”

*Changed*

12. Page 5, line 33: Change "sequences that 500 bp" to "sequences that are 500 bp”

*This sentence has been revised*

13. Page 6, line 25: Please clarify if the figure is Fig. 1E or Fig 2E.

*It is Fig. 2E*

14. Page 6, lines 47-48: Change the three "totally" to “total"

*Changed*

15. Page 6, line 60 and Page 7, line 2: Change "at the presence" to "in the presence”

*Changed*

16. Page 7, line 11: Change "flanked LU24 is" to "flanking LU24 are”

*Changed*

17. Page 7, line 11 and Page 10, line 23: Change "can not" to “cannot"

*Changed*

18. Page 9, line 2: Change "out of the totally" to "out of the total”

*Changed*

19. Page 10, line 11: Change "URA3 integrated" to “URA3-integrated"

*Changed*

20. Page 10, line 17: Change "chose" to “choice"

*Changed*

21. Page 10, line 55: Change "sequences been deleted" to "sequences deleted”

*Changed*

22. Page 10, line 55: Change "we shown" to "we have shown”

*Changed*

23. Page 12, line 40: Please revise "sub" to a more suitable term

*➢ This sentence has been revised to “1/10 of the overnight culture was added into 5 mL of new medium with 2% w/v raffinose and 0.1% w/v glucose”*

24. Page 14, line 30: Please replace "XXX" with the accession number

*➢ Accession number was added.*

25. Page 16, line 21: Change "centromere containing" to "centromere-containing" throughout the manuscript (Fig. S2, S3 and S10 too)

*➢ Changed*

26. Figures 2A, 2B, 3B, 4A: Please change cyan to another colour for the centromere-containing LU. Cyan does not stand out well next to gray.

*➢ It has been changed to brown.*

**Second round of review**

**Reviewer 1**

The authors have addressed my comments and concerns and have significantly improved the manuscript in terms of written content and experimental evidence. I am happy for this work to be published.

**Reviewer 2**

The authors have addressed my comments and I recommend acceptance of the manuscript.
